# A multi-level dynamic sample augmentation and heterogeneous feature fusion framework for intelligent intrusion detection

**DOI:** 10.1038/s41598-026-50301-y

**Published:** 2026-04-30

**Authors:** Ben Li, Wenwen Sun, Dongdong Su, Qiong Li, Dongdong Zhang, Dong Li, Jingnan Huang

**Affiliations:** 1 State Grid Xinjiang Electric Power Co., Ltd. Bazhou Power Supply Company, Bazhou, 841000 China; 2 School of Mechanical and Electrical Engineering, University of Electronic Science and Technology of China, Chengdu, 611731 China

**Keywords:** Engineering, Mathematics and computing

## Abstract

This paper proposes a hybrid intrusion detection framework that integrates dynamic sample augmentation and heterogeneous feature fusion to address class imbalance and temporal complexity in network traffic. At the data level, an ADASYN–FLS–SMOTEBoost strategy combines adaptive oversampling with Focal Loss–Softmax (FLS) weighting to generate more representative minority samples. At the feature level, a multi-branch CNN with FLS-guided dynamic fusion adaptively integrates heterogeneous traffic features. A hierarchical LSTM (HLSTM) is further employed to capture both short-term bursts and long-term temporal dependencies, enhancing temporal modeling capability. To optimize performance, an improved Grey Wolf Optimizer (IGWO) with chaotic initialization and random-walk learning rate adjustment is introduced for efficient hyperparameter tuning. Experimental results on the NSL-KDD dataset show that the proposed method significantly outperforms existing machine learning and deep learning baselines, achieving up to 10% improvement in Macro-F1 and markedly higher recall for rare attack types such as R2L and U2R, while maintaining stable overall accuracy. The proposed framework demonstrates strong robustness, interpretability, and generalization in complex and imbalanced network intrusion scenarios.

## Introduction

With the continuous advancement of informatization and digitalization, the network has become a critical infrastructure for social operation and economic development. At the same time, the frequency and complexity of cyberattacks are on the rise. From massive Denial of Service (DoS) attacks to intrusions and data exfiltration targeting enterprise databases, and even Advanced Persistent Threats (APTs) characterized by long latency and high destructive power, cybersecurity incidents not only threaten personal privacy and financial security but also pose severe risks to national critical information infrastructure^1^. Therefore, how to effectively detect and defend against diverse network intrusions has become a key challenge in cyberspace security research.

Among various security defense technologies, Network Intrusion Detection Systems (NIDS) serve as one of the primary lines of defense. By analyzing network traffic in real time, NIDS can identify potential attacks and provide timely responses for security management^2^. The core of NIDS lies in the design and optimization of classification algorithms. Traditional methods have relied heavily on feature engineering and statistical machine learning algorithms such as decision trees, support vector machines (SVM), and naïve Bayes^3^. Although these methods achieved good performance on early datasets, they often struggle with generalization in large-scale, high-dimensional, and dynamically evolving network environments, and their feature extraction depends significantly on human expertise^4–6^.

In recent years, the emergence of deep learning has offered new opportunities for intrusion detection. Convolutional Neural Networks (CNNs) can automatically extract high-level semantic features^7^, while Recurrent Neural Networks (RNNs) and their variants, such as Long Short-Term Memory (LSTM), are capable of capturing temporal dependencies^8^. These models have demonstrated superior detection accuracy compared to traditional machine learning approaches on benchmark datasets such as KDDCup99, NSL-KDD, and CICIDS^9^. However, deep learning models in NIDS still face multiple challenges, particularly in the following areas.

First, the issue of severe class imbalance persists. In real-world network traffic, normal samples dominate, while rare attack classes (e.g., R2L, U2R) account for only a small fraction^10^. Without proper handling, deep models tend to bias toward majority classes, leading to poor detection performance on rare attacks. To address this, researchers have explored undersampling, oversampling, and cost-sensitive learning techniques. For instance, Synthetic Minority Oversampling Technique (SMOTE) and its improved variant ADASYN (Adaptive Synthetic Sampling) generate synthetic minority samples to mitigate imbalance^11^. However, most existing oversampling techniques only consider sample space distribution, lacking dynamic coupling with model learning difficulty, thus limiting their effectiveness in enhancing boundary discrimination.

Second, the heterogeneity of features and the challenge of multi-source fusion remain significant. Network traffic features typically include protocol fields, statistical behaviors, temporal patterns, and content-based attributes. These dimensions differ semantically, and naive concatenation may cause interference, thereby reducing model efficiency^12^. To alleviate this, multi-branch CNN architectures (Multi-CNN) have been proposed, where different feature groups are modeled separately. Yet, adaptive fusion of such heterogeneous branches is still challenging. Current methods often rely on static weights or simple concatenation, lacking mechanisms to dynamically adjust feature contributions at the sample level.

Third, the complexity of temporal dependencies poses another challenge. Some attacks, such as DoS, exhibit short-term bursts, whereas others, such as R2L and APT, evolve gradually and remain stealthy over longer timescales. A single-layer LSTM suffers from gradient vanishing and excessive forgetting when handling long sequences, making it difficult to capture both short- and long-term patterns effectively. To address this, researchers have introduced Hierarchical LSTM (HLSTM), which performs intra-segment and inter-segment modeling to capture multi-scale temporal patterns^13^. However, the performance of HLSTM largely depends on segmentation strategies and hyperparameter configurations; without careful tuning, its effectiveness may be limited.

Fourth, hyperparameter optimization remains a bottleneck^14,15^. The performance of deep learning models is highly sensitive to both architectural parameters (e.g., kernel size, number of layers, hidden units) and training parameters (e.g., learning rate, batch size)^16^. Traditional grid search and random search are inefficient, while Bayesian optimization becomes computationally expensive in high-dimensional spaces. Recently, swarm intelligence algorithms, such as Particle Swarm Optimization (PSO) and Grey Wolf Optimizer (GWO), have been applied to hyperparameter selection, but they still face issues such as slow convergence and susceptibility to local optima^17,18^.

In summary, existing studies on intrusion detection have made substantial progress, yet several limitations remain: (1) oversampling methods fail to integrate both sample space difficulty and model learning difficulty; (2) feature fusion mechanisms rely primarily on static weighting, lacking sample-level adaptivity; (3) single temporal models struggle to simultaneously capture both short- and long-term attack patterns; and (4) hyperparameter optimization suffers from trade-offs between efficiency and accuracy.

To address these issues, the major contributions of this paper are summarized as follows^19–23^:

- Propose an ADASYN-FLS-SMOTEBoost strategy that incorporates spatial difficulty weights and Focal Loss probability calibration to emphasize borderline and hard-to-learn minority samples.
- Design a Multi-CNN architecture with an adaptive fusion mechanism, enabling the dynamic integration of heterogeneous traffic features through sample-level weighting.
- Develop a hierarchical HLSTM (Multi-CNN-HLSTM) to simultaneously capture multi-scale temporal dependencies, preserving short-term burst features while extracting long-term latent attack chains.
- Apply an Improved Grey Wolf Optimizer (IGWO) enhanced by chaotic initialization and random-walk learning to automatically tune hyperparameters, significantly improving global search capability and convergence stability.

The remainder of this paper is structured as follows. Section 2 introduces the theoretical foundations and related work. Section 3 details the proposed model framework, including ADASYN–FLS–SMOTEBoost, the Multi-CNN adaptive fusion mechanism, the HLSTM architecture, and IGWO optimization. Section 4 describes the experimental design, including datasets, evaluation metrics, and baseline comparisons. Section 5 presents and analyzes the experimental results. Section 6 concludes the study and discusses future research directions.

## Theoretical background

### Class imbalance and advanced oversampling strategies

In Network Intrusion Detection (NID) tasks, datasets commonly exhibit a pronounced class imbalance, where the number of normal traffic samples is substantially higher than that of various attack samples. In some cases, certain attack types account for only a negligible proportion of the total sample volume^24^. When conventional supervised learning models are directly applied, the learning process tends to be dominated by majority-class samples, resulting in a significant decline in the detection rate of minority-class attacks and, consequently, a degradation of the overall system robustness.

Let $\:{N}_{\mathrm{m}\mathrm{a}\mathrm{j}}$ and $\:{N}_{\mathrm{m}\mathrm{i}\mathrm{n}}$ denote the numbers of majority-class and minority-class samples in the training set, respectively. The imbalance ratio can then be expressed as:

1 $$\:\begin{array}{c}r=\frac{{N}_{\mathrm{m}\mathrm{a}\mathrm{j}}}{{N}_{\mathrm{m}\mathrm{i}\mathrm{n}}}\end{array}$$

When $\:r\gg\:1$, the optimization objective of the model tends to favor the majority class, resulting in severely biased predictions. Therefore, mitigating the impact of imbalanced data on classification performance has become a critical challenge in intrusion detection research.

To address the aforementioned issue, various resampling strategies have been proposed in the research community. The most representative approach is SMOTE (Synthetic Minority Oversampling Technique). SMOTE generates new minority samples by performing linear interpolation between a minority sample and its $\:k$ nearest neighbors. The generation process can be expressed as follows^25^:

2 $$\:\begin{array}{c}\stackrel{\sim}{x}={x}_{i}+\delta\:\cdot\:\left({x}_{zi}-{x}_{i}\right),\:\delta\:\sim\:U\left(0,1\right)\end{array}$$

where, $\:{x}_{i}$ denotes an original minority-class sample, and $\:{x}_{zi}$ represents a minority sample within its neighborhood. SMOTE expands the distribution space of minority samples to some extent; however, its uniform sample generation mechanism often overlooks the distribution characteristics of boundary regions or hard-to-classify samples.

To overcome this limitation, ADASYN (Adaptive Synthetic Sampling Approach) introduces an adaptive difficulty metric mechanism. In ADASYN, for each minority-class sample $\:{x}_{i}$, the proportion of majority-class samples within its k-nearest neighborhood is calculated as $\:{r}_{i}$, and the corresponding weights are obtained through normalization.

3 $$\:\begin{array}{c}{g}_{i}=\frac{{r}_{i}}{{\sum\:}_{j}{r}_{j}}\end{array}$$

Given a total number of generated samples $\:G$, the number of synthetic samples generated for each instance is defined as:

4 $$\:\begin{array}{c}{G}_{i}={g}_{i}\cdot\:G\end{array}$$

In this way, ADASYN can generate more samples in the classification boundary regions, thereby improving the model’s discriminative capability for minority classes.

### Focal loss for hard example learning

In scenarios with extreme class imbalance, the traditional cross-entropy loss is often dominated by a large number of easily classified majority-class samples, making it difficult to guide the model to learn critical minority-class patterns. To address this issue, Focal Loss was proposed^26,27^ to automatically adjust the loss contribution of different samples. The formulation is given as follows:

5 $$\:\begin{array}{c}{\mathcal{L}}_{\mathrm{F}\mathrm{L}}=-{\alpha\:}_{t}{\left(1-{p}_{t}\right)}^{\gamma\:}log\left({p}_{t}\right)\end{array}$$

where, $\:{p}_{t}$ denotes the predicted probability of the true class, $\:\gamma\:\ge\:0$ is the focusing parameter, and $\:{\alpha\:}_{t}$ represents the class-balancing coefficient. When $\:{p}_{t}$ is large, the corresponding loss value is significantly reduced, thereby diminishing the influence of easily classified samples; conversely, hard-to-classify samples are assigned greater weights.

Building upon this concept, researchers further proposed the Focal Loss–Softmax (FLS) framework, which integrates the Softmax output with the difficulty-adjustment mechanism of Focal Loss to guide branch fusion and sample weighting^28,29^. This strategy enables the model to dynamically focus on minority and hard samples with ambiguous decision boundaries, thereby enhancing overall detection accuracy and stability.

### Synergistic mechanism of boosting and SMOTEBoost

The Boosting method enhances classification performance by iteratively training multiple weak classifiers and integrating them through weighted aggregation. Among them, the sample weight updating strategy of AdaBoost is defined as:

6 $$\:\begin{array}{c}{D}_{t+1}\left(i\right)=\frac{{D}_{t}\left(i\right)\mathrm{exp}\left(-{\alpha\:}_{t}{y}_{i}{h}_{t}\left({x}_{i}\right)\right)}{{Z}_{t}}\end{array}$$

where, $\:{D}_{t}\left(i\right)$ denotes the sample weight at the *t*-th iteration, and $\:{\alpha\:}_{t}$ is related to the classifier’s error rate. This mechanism enables subsequent weak classifiers to place greater emphasis on samples previously misclassified.

In the context of class imbalance, SMOTEBoost^30,31^ integrates SMOTE with Boosting: before each iteration, SMOTE is applied to oversample the minority class, thereby dynamically balancing the training set. This mechanism effectively mitigates the adverse impact of class imbalance on ensemble learning. However, SMOTEBoost still suffers from the “average generation” issue during synthetic sample creation, lacking focused attention on hard-to-classify and borderline minority samples.

To address this limitation, recent studies have gradually incorporated mechanisms such as ADASYN and Focal Loss, leading to the development of the ADASYN–FLS–SMOTEBoost framework^32,33^, which enables targeted modeling of difficult minority-class instances.

### Multi-path convolutional networks and feature group learning

Network traffic data comprises multi-source heterogeneous features, such as protocol fields, content statistics, session behaviors, and time-series characteristics. The correlations among these features vary significantly, and directly concatenating them as input may lead to inefficient model learning. To address this issue, a Multi-Channel Convolutional Neural Network (Multi-CNN) has been proposed^34,35^, in which independent convolutional branches are designed for different feature groups, enabling the model to capture local patterns within each group. The computation of a single convolutional layer can be expressed as:

7 $$\:\begin{array}{c}{\left(y\times\:w\right)}_{t}=\sum\:_{k=1}^{K}{w}_{k}{x}_{t+k-1}+b\end{array}$$

After pooling and activation functions (such as ReLU or ELU), the extracted representations can be further integrated. In the multi-branch architecture, the outputs of different branches can be adaptively combined through the Softmax–FLS weighted fusion mechanism:

8 $$\:\begin{array}{c}{w}_{i}=\frac{\mathrm{s}\mathrm{o}\mathrm{f}\mathrm{t}\mathrm{m}\mathrm{a}\mathrm{x}\left({s}_{i}\right)\hspace{0.17em}(1-{p}_{i}{)}^{\gamma\:}}{\sum\:_{j}\mathrm{s}\mathrm{o}\mathrm{f}\mathrm{t}\mathrm{m}\mathrm{a}\mathrm{x}\left({s}_{j}\right)\hspace{0.17em}(1-{p}_{j}{)}^{\gamma\:}},\:z=\sum\:_{i}{w}_{i}{f}_{i}\end{array}$$

where, $\:{s}_{i}$ denotes the score of the *i*-th branch, and $\:{p}_{i}$ represents the predicted probability from that branch. This approach emphasizes the most discriminative feature group for the current sample, thereby enhancing the model’s robustness and interpretability.

### Temporal modeling with hierarchical LSTM (HLSTM)

Network intrusion behaviors can exhibit either short-term bursts (e.g., DoS attacks) or long-term latent characteristics (e.g., R2L and U2R attacks). Single-layer LSTMs often suffer from vanishing gradients or excessive forgetting when modeling long sequences. To address this, researchers proposed the Hierarchical LSTM (HLSTM)^36,37^, which achieves multi-scale temporal modeling through a short-term within segments + long-term across segments architecture. The state update equations of the standard LSTM are as follows:

9 $$\:\begin{array}{c}\begin{array}{c}{i}_{t}=\sigma\:\left({W}_{i}{x}_{t}+{U}_{i}{h}_{t-1}+{b}_{i}\right),\\\:{f}_{t}=\sigma\:\left({W}_{f}{x}_{t}+{U}_{f}{h}_{t-1}+{b}_{f}\right),\\\:{o}_{t}=\sigma\:\left({W}_{o}{x}_{t}+{U}_{o}{h}_{t-1}+{b}_{o}\right),\\\:{\stackrel{\sim}{c}}_{t}=\mathrm{tanh}\left({W}_{c}{x}_{t}+{U}_{c}{h}_{t-1}+{b}_{c}\right),\\\:{c}_{t}={f}_{t}\odot\:{c}_{t-1}+{i}_{t}\odot\:{\stackrel{\sim}{c}}_{t},\\\:{h}_{t}={o}_{t}\odot\:\mathrm{tanh}\left({c}_{t}\right).\end{array}\end{array}$$

In the HLSTM, the lower-level LSTM models short time windows to obtain segment vectors $\:{s}_{j}$. Subsequently, the higher-level LSTM performs global modeling over the sequence $\:\left\{{s}_{j}\right\}$, with an attention mechanism introduced when necessary:

10 $$\:\begin{array}{c}{\alpha\:}_{j}=\frac{\mathrm{exp}\left({v}^{\top\:}\mathrm{tanh}\left(W{s}_{j}\right)\right)}{{\sum\:}_{k}\mathrm{e}\mathrm{x}\mathrm{p}\left({v}^{\top\:}\mathrm{tanh}\left(W{s}_{k}\right)\right)},\:r=\sum\:_{j}{\alpha\:}_{j}{s}_{j}\end{array}$$

This structure preserves short-term burst information while enhancing the ability to capture long-term dependencies, making it particularly suitable for detecting complex attack behaviors.

### Swarm intelligence optimization and improved grey wolf optimizer (IGWO)

The performance of deep learning models largely depends on the configuration of hyperparameters (such as convolution kernel size, learning rate, and LSTM hidden layer dimension). Traditional grid search and random search methods are inefficient and prone to local optima. The Grey Wolf Optimizer (GWO)^38,39^ is a population intelligence-based global optimization algorithm that simulates the hunting behavior of grey wolves to perform search and update operations. Its position update equation is as follows:

11 $$\:\begin{array}{c}{X}_{\mathrm{n}\mathrm{e}\mathrm{w}}=\frac{{X}_{1}+{X}_{2}+{X}_{3}}{3},\:{X}_{1}={X}_{\alpha\:}-{A}_{1}\cdot\:\left|{C}_{1}{X}_{\alpha\:}-X\right|\end{array}$$

where, $\:{X}_{\alpha\:}$, $\:{X}_{\beta\:}$, and $\:{X}_{\delta\:}$ denote the positions of the top three individuals in the population, and $\:A$ and $\:C$ are dynamic coefficients.

To enhance convergence speed and global search capability, this paper adopts the Improved Grey Wolf Optimizer (IGWO)^42^. Chaotic mapping is introduced during the initialization phase to increase solution diversity, and a random perturbation factor is incorporated during iterations to prevent premature convergence. IGWO employs the model’s overall performance on the validation set, such as Macro-F1 and AUPRC be as the fitness function, thereby enabling adaptive search and optimization of hyperparameters.

### Recent related works in intrusion detection

Recent advancements in NIDS have explored diverse methodologies ranging from unsupervised clustering to advanced deep learning frameworks. For instance, Prasad et al. proposed a clustering method based on unsupervised feature selection and cluster center initialization^40^. Strengths: This method effectively handles unlabeled data, mitigates computational complexity, and forms arbitrary-shaped micro-clusters to detect new attacks and accommodate density variations. Limitations: The reliance on spatial distance for micro-clustering may face challenges when applied to extremely imbalanced datasets with highly overlapping decision boundaries. To address evaluation inconsistencies, Prasad et al. later introduced an intelligent intrusion detection and performance reliability evaluation mechanism^41^. Strengths: It utilizes a fuzzy logic system to strictly compute and evaluate the reliability of the detection scheme, offering a robust quantitative metric beyond standard accuracy. Limitations: As explicitly noted in their study, an imbalanced sample ratio inherently causes a trade-off where increasing one statistical performance metric inevitably decreases another, highlighting the persistent bottleneck of class imbalance in NIDS. The framework proposed in this paper specifically targets this limitation by integrating dynamic sample augmentation and feature fusion to balance comprehensive performance metrics.

### Multi-level adaptive sample generation framework: a dynamic augmentation mechanism based on collaborative weight optimization

#### Adaptive weight initialization for minority classes based on ADASYN distribution learning

ADASYN adaptively adjusts the number of synthetic minority samples based on the difficulty of their distribution in the feature space. Specifically, it generates more synthetic samples in regions where minority samples are sparsely distributed or located near the decision boundary with the majority class, while generating fewer or no new samples in easily separable regions. By prioritizing difficult samples and assigning less attention to easy ones, this strategy enables the classifier to better learn the discriminative characteristics of the minority class during training.

Formally, let the set of minority-class samples in the training set be $\:{\mathcal{S}}_{\mathrm{m}\mathrm{i}\mathrm{n}}=\{{x}_{1},{x}_{2},\dots\:,{x}_{{N}_{\mathrm{m}\mathrm{i}\mathrm{n}}}\}$, and the set of majority-class samples be $\:{\mathcal{S}}_{\mathrm{m}\mathrm{a}\mathrm{j}}$. The class imbalance ratio can be expressed as:

12 $$\:\begin{array}{c}r=\frac{{N}_{\mathrm{m}\mathrm{a}\mathrm{j}}}{{N}_{\mathrm{m}\mathrm{i}\mathrm{n}}}\end{array}$$

where $\:{N}_{\mathrm{m}\mathrm{a}\mathrm{j}}$ and $\:{N}_{\mathrm{m}\mathrm{i}\mathrm{n}}$ denote the number of majority-class and minority-class samples, respectively. If $\:r>{r}_{\mathrm{t}\mathrm{h}}$ (a predefined threshold), an oversampling strategy is triggered. For each minority-class sample $\:{x}_{i}$, the proportion of majority-class samples among its k-nearest neighbors in the feature space is computed as:

13 $$\:\begin{array}{c}{r}_{i}=\frac{{{\Delta\:}}_{i}}{k}\end{array}$$

where $\:{{\Delta\:}}_{i}$ denotes the number of majority-class samples among the k-nearest neighbors of $\:{x}_{i}$. Intuitively, a higher proportion of majority-class samples in the neighborhood of $\:{x}_{i}$ leads to a larger $\:{r}_{i}$, indicating that the sample is more difficult to learn. Subsequently, the values $\:{r}_{i}$ for all minority-class samples are normalized to obtain a weight distribution:

14 $$\:\begin{array}{c}{w}_{i}=\frac{{r}_{i}}{\sum\:_{j=1}^{{N}_{\mathrm{m}\mathrm{i}\mathrm{n}}}{r}_{j}},\:i=1,2,\dots\:,{N}_{min}\end{array}$$

The weight $\:{w}_{i}$ represents the proportion of synthetic samples to be generated for each minority-class sample during the sampling process. The total number of synthetic samples to be generated is given by:

15 $$\:\begin{array}{c}G=\left({N}_{\mathrm{m}\mathrm{a}\mathrm{j}}-{N}_{\mathrm{m}\mathrm{i}\mathrm{n}}\right)\cdot\:\beta\:\end{array}$$

where $\:\beta\:\in\:[0,1]$ is a sampling intensity parameter that controls the degree to which the disparity between the minority and majority classes is reduced. For a given minority sample $\:{x}_{i}$, the number of synthetic samples to be generated is:

16 $$\:\begin{array}{c}{g}_{i}={w}_{i}\cdot\:G\end{array}$$

The generation process is similar to SMOTE, i.e., synthetic samples are created by interpolating between $\:{x}_{i}$ and its minority-class nearest neighbors:

17 $$\:\begin{array}{c}{x}_{\mathrm{n}\mathrm{e}\mathrm{w}}={x}_{i}+\lambda\:\cdot\:\left({x}_{\mathrm{n}\mathrm{n}}-{x}_{i}\right)\end{array}$$

where $\:{x}_{\mathrm{n}\mathrm{n}}$ denotes a minority-class nearest neighbor of $\:{x}_{i}$, and $\:\lambda\:\sim\:U(0,1)$.

In network traffic anomaly detection, malicious traffic often exhibits the following two characteristics: sparse distribution and ambiguous boundaries. Sparse distribution means that samples of certain attack types are extremely few in the original dataset and are easily ignored during training; ambiguous boundaries manifest as the similarity between certain attack behaviors and normal traffic in statistical features, making them hard to distinguish. Traditional uniform oversampling methods generate new samples evenly across all minority-class instances and cannot provide extra support to these difficult regions, thus performing poorly in boundary discrimination. The advantage of ADASYN in this scenario is: generating a small number of samples in easy-to-classify regions to avoid ineffective gains for the model and reduce training overhead from redundant data; focusing on generating samples in difficult-to-classify regions to enhance the classifier’s discriminative ability for boundary samples and improve the model’s learning effectiveness in ambiguous regions; dynamic sample weight guidance: through dynamic adjustment of the weight distribution $\:{w}_{i}$, the newly generated samples better conform to the true decision boundary, alleviating the bias caused by extreme class imbalance. For example, when modeling the NSL-KDD dataset, the number of samples for certain DoS or Web attack types is far smaller than that of normal traffic. After applying ADASYN, the model generates additional synthetic samples in the boundary regions of these attacks, thereby improving the detector’s sensitivity to DoS or Web attacks, rather than simply increasing the number of all minority-class samples.

#### Dynamic reweighting of hard samples based on FLS probability calibration

Building on ADASYN, this work further introduces the FLS (Focal Loss–Softmax) probability distribution weight adjustment mechanism to enhance the learning of hard-to-classify minority samples. In traditional ADASYN, the computation of sample weights primarily relies on the majority-class ratio $\:{r}_{i}$ within a sample’s neighborhood, which reflects the “learning difficulty” of that sample in feature space. However, relying solely on spatial neighborhood distribution has limitations: it does not directly capture the classification model’s difficulty in discriminating the sample during training.

For instance, some minority samples may be evenly distributed in feature space yet are still misclassified as majority class by the model; conversely, certain boundary samples may appear complex in their neighborhood but are easily classified correctly. Thus, neighborhood-based weighting alone cannot fully characterize sample difficulty.

To address this issue, we introduce a Focal Loss–based weight correction mechanism. Originally proposed in object detection, Focal Loss reduces the loss contribution of easily classified samples while increasing that of hard samples, thereby guiding the model to focus on difficult cases. This principle aligns well with ADASYN’s strategy of generating more synthetic samples in hard regions, making it suitable for integration into the oversampling weight update process.

Formally, let the prediction probability of the classification model on sample $\:{x}_{i}$ be $\:{p}_{i}\in\:[0,1]$, and the true label be $\:{y}_{i}\in\:\{0,1\}$. The standard cross-entropy loss is defined as:

18 $$\:\begin{array}{c}{\mathcal{L}}_{CE}=-{y}_{i}log\left({p}_{i}\right)\end{array}$$

Focal Loss introduces two adjustment factors on this basis: a hard-example modulation factor $\:(1-{p}_{i}{)}^{\gamma\:}$, where $\:\gamma\:>0$ is the focusing parameter. When $\:{p}_{i}$ is closer to 1 (i.e., the sample is easy to classify), this factor approaches 0, reducing the sample’s loss contribution; when $\:{p}_{i}$ is smaller (i.e., the sample is hard to classify), the factor approaches 1, amplifying the loss contribution. A class-balancing factor $\:\alpha\:$ is used to control the weight allocation between the minority and majority classes. The Focal Loss is then defined as:

19 $$\:\begin{array}{c}{\mathcal{L}}_{\mathrm{F}\mathrm{L}}=-\alpha\:\cdot\:{\left(1-{p}_{i}\right)}^{\gamma\:}{\cdot\:y}_{i}\cdot\:log\left({p}_{i}\right)\end{array}$$

In the proposed framework, we leverage the gradient information from Focal Loss to assign a new weight to each sample:

20 $$\:\begin{array}{c}{w}_{i}^{\mathrm{F}\mathrm{L}\mathrm{S}}=\alpha\:\cdot\:{\left(1-{p}_{i}\right)}^{\gamma\:}\end{array}$$

where $\:{w}_{i}^{\mathrm{F}\mathrm{L}\mathrm{S}}$ directly reflects the learning difficulty of sample $\:{x}_{i}$ during model training. Subsequently, the spatial neighborhood weight $\:{w}_{i}$ from ADASYN is fused with the FLS weight $\:{w}_{i}^{\mathrm{F}\mathrm{L}\mathrm{S}}$:

21 $$\:\begin{array}{c}{w}_{i}^{\mathrm{f}\mathrm{i}\mathrm{n}\mathrm{a}\mathrm{l}}=\lambda\:\cdot\:{w}_{i}+\left(1-\lambda\:\right)\cdot\:{w}_{i}^{\mathrm{F}\mathrm{L}\mathrm{S}},\:\lambda\:\in\:\left[0,1\right]\end{array}$$

By incorporating FLS weights, the resulting weight distribution accounts for both the spatial distribution complexity of the samples and the model’s prediction difficulty, yielding a more comprehensive weighting scheme.

In IoT intrusion detection, malicious traffic typically exhibits multiple complex patterns. For instance, some DoS attack samples highly overlap with normal traffic in statistical features, causing classifiers to easily misclassify them as normal; meanwhile, certain Web attack samples, although clearly separable in the feature space, are too scarce in number for the model to learn effective patterns during training. Relying solely on ADASYN’s neighborhood information is insufficient to fully address these scenarios. By introducing FLS weights, the model can automatically identify which minority-class samples are more prone to misclassification during training and thus allocate additional attention to these hard-sample regions in subsequent synthetic generation. For example, in experiments on the NSL-KDD dataset, we observed that DoS attack samples did not receive prominent weights under ADASYN sampling, but their importance significantly increased after FLS weight calibration. The combined weight $\:{w}_{i}^{\mathrm{f}\mathrm{i}\mathrm{n}\mathrm{a}\mathrm{l}}$ enables these samples to be prioritized in subsequent SMOTEBoost iterations, thereby effectively improving the classifier’s detection rate.

#### Dynamic sample evolution mechanism via SMOTEBoost–ADASYN–FLS collaborative ensemble

After the initial weight assignment by ADASYN and hard-sample correction by FLS, the importance distribution of minority-class samples has been reasonably optimized. However, a single oversampling step remains limited: the generated data are static and cannot be dynamically adjusted according to the evolving discriminative capability of the model during training. This implies that, even with optimized sample weights, the model may gradually drift toward the majority class in subsequent iterative training, causing the initially enhanced sensitivity to minority classes to diminish over time.

To address this issue, this paper further incorporates the SMOTEBoost ensemble enhancement mechanism, integrating synthetic minority-class sampling with Boosting-based ensemble learning to achieve collaborative optimization between sample generation and classifier training.

In the proposed method, SMOTEBoost does not perform sampling directly on the original minority-class samples. Instead, it uses the final weight distribution $\:{w}_{i}^{\mathrm{f}\mathrm{i}\mathrm{n}\mathrm{a}\mathrm{l}}$, optimized in the previous two steps (ADASYN and FLS), as input. Specifically, in each iteration, the synthetic samples generated by SMOTE are no longer based on uniform weights, but on:

22 $$\:\begin{array}{c}{w}_{i}^{\mathrm{f}\mathrm{i}\mathrm{n}\mathrm{a}\mathrm{l}}=\lambda\:\cdot\:{w}_{i}+\left(1-\lambda\:\right)\cdot\:{w}_{i}^{\mathrm{F}\mathrm{L}\mathrm{S}}\end{array}$$

where $\:{w}_{i}$ originates from ADASYN’s spatial neighborhood weight, $\:{w}_{i}^{\mathrm{F}\mathrm{L}\mathrm{S}}$ comes from Focal Loss calibration, and $\:\lambda\:$ is the fusion coefficient.

This fusion strategy offers two key advantages. First, in terms of dynamism, due to the iterative mechanism of SMOTEBoost being tightly coupled with classifier performance, sample weights are not fixed once at the beginning of training. Instead, they are updated after each iteration based on the classifier’s current discriminative performance. Consequently, the distribution of synthetic samples continuously adapts throughout training, always focusing on the model’s weakest regions. This ensures that newly generated samples genuinely provide supplementary information and avoids the degradation commonly observed in traditional static oversampling methods during later training stages.

Second, regarding targeted emphasis, the incorporation of FLS ensures that hard-to-classify minority samples receive higher attention in weight allocation. By suppressing the influence of easy samples through the focusing factor, FLS directs the model’s limited learning capacity toward more challenging regions. When this weight calibration is combined with Boosting’s iterative updating, the classifier progressively strengthens its learning of critical boundary areas and high-risk minority samples across iterations, establishing a hard-sample-first training trajectory. This significantly enhances the model’s robustness and sensitivity under extreme class imbalance.

Therefore, the proposed ADASYN–FLS–SMOTEBoost framework is not a simple stacking of three methods, but rather a holistic upgrade across the entire pipeline—from static weighted sampling (driven by ADASYN’s spatial neighborhood information) to dynamic iterative sampling (guided by FLS-based correction and weight updating), and finally to collaborative optimization between sampling and model training (enabled by SMOTEBoost’s ensemble iteration). This progressive design preserves spatial distribution information, incorporates feedback from model learning difficulty, and continuously refines the sample distribution through iterative training, thereby achieving simultaneous improvements in detection performance and generalization capability. Compared with conventional approaches, the framework better aligns with the dynamic evolution characteristics of network traffic and is particularly well-suited for complex scenarios such as IoT intrusion detection, where extreme class imbalance and unknown attack patterns coexist.

#### Overall pipeline and collaborative optimization process

**Fig. 1:**
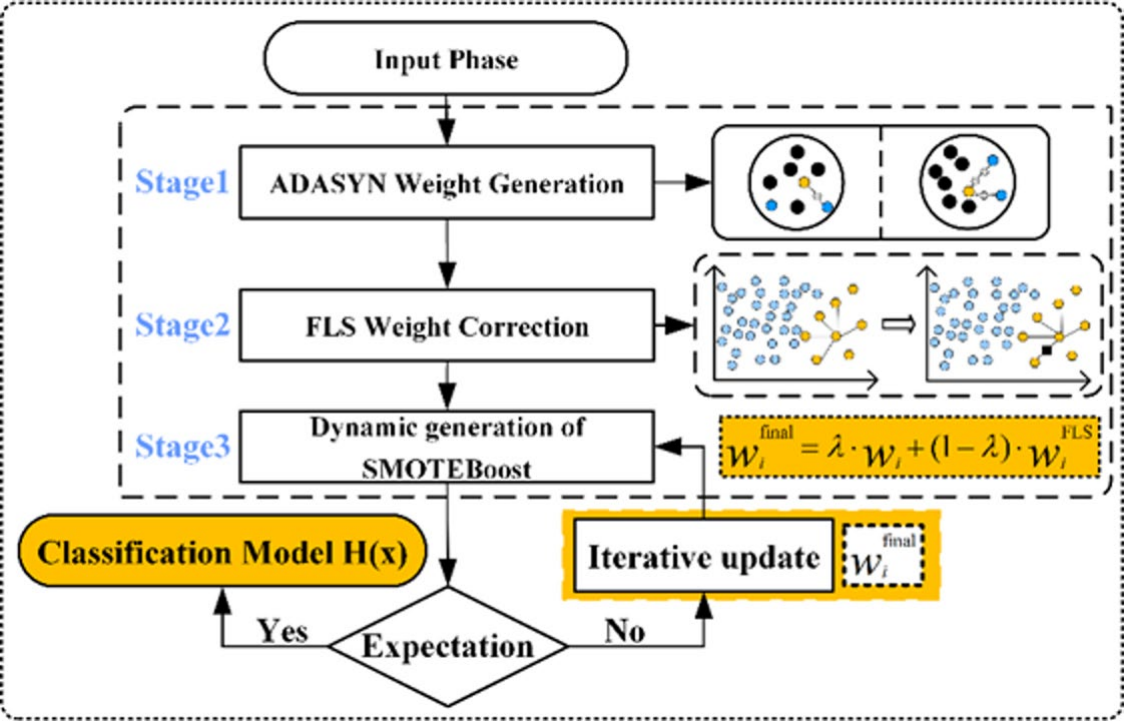
ADASYN–FLS–SMOTEBoost integrated dynamic sample generation method.

To more systematically elucidate the proposed ADASYN–FLS–SMOTEBoost integrated dynamic sample generation method, this section provides a logical textual description and further presents a corresponding flowchart. As shown in Fig. 1, the flowchart illustrates the interconnections and information flow among the sub-modules at a macro level, offering a clear and intuitive overview of the overall framework proposed in this section.

**Table:** 

Step1: Input Stage: Receive the original imbalanced dataset $\:\mathcal{D}=\left\{\right({x}_{i},{y}_{i}){\}}_{i=1}^{N}$, where $\:{y}_{i}\in\:\{0,1\}$ and $\:{N}_{\mathrm{m}\mathrm{a}\mathrm{j}}\gg\:{N}_{\mathrm{m}\mathrm{i}\mathrm{n}}$. Initialize the Boosting sample weights as $\:{D}_{1}\left(i\right)=\frac{1}{N}$.
Step2: ADASYN Weight Generation: For each minority-class sample, compute the neighborhood difficulty coefficient $\:{r}_{i}$ and obtain the initial spatial weight distribution $\:{w}_{i}$.
Step3: FLS Weight Calibration and Dynamic Probability Derivation. Combine the model’s predicted probability $\:{p}_{i}$ with Focal Loss to update the hard-sample weight $\:{w}_{i}^{\mathrm{F}\mathrm{L}\mathrm{S}}$, and fuse the two weights as: $\:{w}_{i}^{\mathrm{f}\mathrm{i}\mathrm{n}\mathrm{a}\mathrm{l}}=\lambda\:\cdot\:{w}_{i}+(1-\lambda\:)\cdot\:{w}_{i}^{\mathrm{F}\mathrm{L}\mathrm{S}}$. Implementation Detail: The prediction probability $\:{p}_{i}$ dynamically reflects the learning difficulty of the current ensemble. Specifically, for the very first iteration ($\:t=1$), $\:{p}_{i}$ is provided by a lightweight warm-up base classifier (a simplified single-branch CNN trained for 3 epochs on the original distribution) to establish an initial hardness prior. For all subsequent boosting iterations ($\:t>1$), $\:{p}_{i}$ is directly derived from the aggregated probability output of the weak learners generated up to iteration $\:t-1$, denoted as $\:{H}_{t-1}\left({x}_{i}\right)=\sum\:_{k=1}^{t-1}{\alpha\:}_{k}{h}_{k}\left({x}_{i}\right)$. This ensures the oversampling mechanism is tightly coupled with the actual blind spots of the dynamically growing ensemble.
Step4: SMOTEBoost Dynamic Generation: In each iteration of Boosting, select high-weight minority-class samples according to $\:{w}_{i}^{\mathrm{f}\mathrm{i}\mathrm{n}\mathrm{a}\mathrm{l}}$ for SMOTE synthesis. The newly generated samples are added to the training set, and the classifier is updated accordingly.
Step5: Iterative Update: Based on the classification error $\:{\epsilon}_{t}$ of the newly trained weak classifier $\:{h}_{t}\left(x\right)$ on the current distribution, update the sample distribution to $\:{D}_{t+1}$ to reinforce the influence of hard-to-classify samples. Repeat the above steps until the number of iterations reaches the preset value $\:T$.
Step6: Output Stage: Aggregate all weak classifiers to form the final classification model $\:H\left(x\right)$.

### Heterogeneous Multi-scale Fusion Network: A Multi-CNN Grouping and Hierarchical HLSTM Architecture for Context-Aware Representation Learning

#### Overall Architecture and Design Principles

**Fig. 2:**
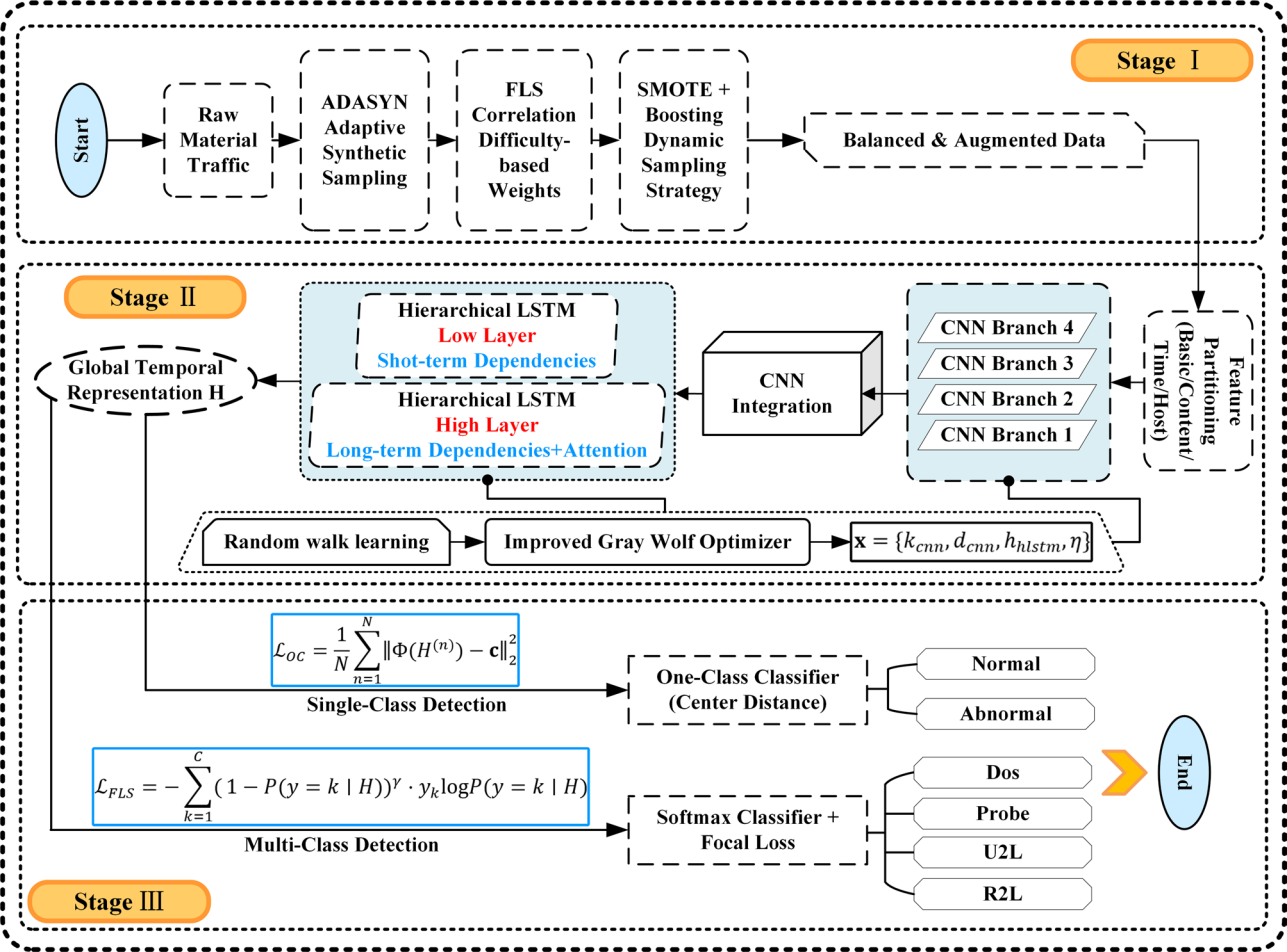
Overall architecture of the proposed method.

The overall pipeline of the proposed method is illustrated in Fig. 2. The design follows the principle of “divide-and-conquer and hierarchical abstraction”: preserving semantic integrity of features at the input stage, enhancing representational capacity through multi-path modeling and dynamic fusion in the intermediate stage, capturing multi-scale temporal dependencies via a hierarchical progressive structure at the temporal level, and enabling targeted detection at the decision stage.

Specifically, the raw traffic is first partitioned into multiple feature subsets based on protocol type, feature correlation, and statistical properties to avoid interference from heterogeneous features. Subsequently, an independent CNN branch is constructed for each subset. ELU activation is employed at the end of each branch to ensure gradient stability, and an FLS fusion module adaptively weights the branches to jointly emphasize salient patterns and hard-to-classify samples.

Furthermore, a hierarchical HLSTM is introduced: the lower layer captures short-term dependencies, while the upper layer models long-term evolution, thereby forming a global temporal representation.

At the decision stage, the framework supports two modes: (1) one-class classification, which performs anomaly detection by minimizing the distance between samples and a learned center; and (2) multi-class classification, which feeds the HLSTM output into a softmax classifier and combines it with FLS loss for fine-grained identification.

The overall framework can be formulated as:

23 $$\:\begin{array}{c}\widehat{y}=F\left(X;{\Theta\:}\right)=D\left(\mathrm{H}\mathrm{L}\mathrm{S}\mathrm{T}\mathrm{M}\left(\mathrm{F}\mathrm{L}\mathrm{S}\right(\left\{\mathrm{C}\mathrm{N}{\mathrm{N}}_{i}\right({X}_{i}\left){\}}_{i=1}^{M}\right))\right)\end{array}$$

where $\:{\Theta\:}$ denotes all network parameters, $\:\mathrm{C}\mathrm{N}{\mathrm{N}}_{i}(\cdot\:)$ represents the *i*-th convolutional branch, $\:\mathrm{F}\mathrm{L}\mathrm{S}(\cdot\:)$ is the multi-branch fusion module, $\:\mathrm{H}\mathrm{L}\mathrm{S}\mathrm{T}\mathrm{M}(\cdot\:)$ denotes the hierarchical temporal modeling component, and $\:D(\cdot\:)$ is the decision module, which can be instantiated as either a one-class discriminator $\:\mathrm{O}\mathrm{C}(\cdot\:)$ or a multi-class softmax classifier.

To avoid local optima caused by manual hyperparameter tuning, this paper further incorporates the Grey Wolf Optimizer (GWO) for adaptive search of key hyperparameters. Specifically, $\:{k}_{\mathrm{c}\mathrm{n}\mathrm{n}}$ denotes the convolutional kernel size, controlling the receptive field of feature extraction; $\:{d}_{\mathrm{c}\mathrm{n}\mathrm{n}}$ represents the depth of the convolutional layers, determining the hierarchy of local feature fusion; $\:{h}_{\mathrm{h}\mathrm{l}\mathrm{s}\mathrm{t}\mathrm{m}}$ is the hidden dimension of HLSTM, reflecting the model’s capacity to capture temporal dependencies; and $\:\eta\:$ is the learning rate, which affects the convergence speed and stability of training.

GWO performs global search in the hyperparameter space, enhanced with a random-walk-based adaptive learning rate to improve exploration capability. This enables the aforementioned hyperparameters to be adaptively adjusted during training, thereby enhancing the robustness and performance of the overall detection framework. Through this combination of hierarchical progressive design and adaptive optimization, the model ensures representational completeness while achieving flexible and efficient intrusion detection under complex network traffic scenarios.

### Multi-branch CNN Architecture and FLS-Guided Dynamic Fusion Strategy

#### Multi-branch CNN Architecture for Feature Grouping

Network traffic data typically comprises tens to hundreds of feature dimensions, including low-level protocol fields (e.g., IP addresses, ports, TCP flags) and high-level statistical features (e.g., connection duration, traffic rate, host-based packet distribution statistics). These features exhibit certain logical correlations but also contain substantial redundancy and noise. Feeding them as a monolithic input not only increases model training complexity but also risks submerging critical features during optimization.

Therefore, guided by established prior knowledge in intrusion detection, this paper partitions the original features into the following four categories, as illustrated in the figure:

- Basic connection features: including protocol type, service type, source/destination ports, etc., which directly reflect fundamental information of an individual connection.
- Content features: such as TCP flags, error codes, and login status, which are closely tied to packet content or protocol semantics.
- Time-based traffic statistical features: statistics computed over a time window, such as the number of connections, packet length distribution, and proportion of anomalous connections, capable of capturing short-term burst behaviors.
- Host-based traffic statistical features: for example, the number of connections, failure ratio, and number of distinct communication peers for a given host over a long period, commonly used to identify persistent attack patterns.

This partitioning strategy offers two key advantages: (1) features within each group share consistent semantics, enabling CNNs to focus more effectively on learning relevant local patterns through convolution; (2) semantic discrepancies across groups are handled via decoupled branches, avoiding artificial spatial correlations that would arise from forcing heterogeneous features into a single 2D matrix representation.

In designing the multi-path CNN architecture, each feature group $\:{X}_{i}\in\:{\mathbb{R}}^{T\times\:{d}_{i}}$ is processed by an independent CNN module for feature extraction. The mapping of the *i*-th branch can be expressed as:

24 $$\:\begin{array}{c}{\widehat{G}}_{i}={g}_{i}\left({X}_{i}\right),\:i=1,2,3,4\end{array}$$

where $\:{g}_{i}(\cdot\:)$ denotes the mapping function of the *i*-th CNN branch, and the output $\:{\widehat{G}}_{i}\in\:{\mathbb{R}}^{T\times\:d}$ is the deep feature representation corresponding to the *i*-th feature group. Taking the *i*-th branch as an example, the convolution operation at time step $\:t$ can be expressed as:

25 $$\:\begin{array}{c}{h}_{i,j}^{t}=\sum\:_{u=1}^{k}{W}_{i,j,u}\cdot\:{x}_{i,t+u-1}+{b}_{i,j},\:j=1,\dots\:,{C}_{i}\end{array}$$

where $\:{W}_{i,j,u}$ denotes the convolutional kernel parameters and $\:{C}_{i}$ is the number of convolutional kernels. After pooling and batch normalization (BN), the output is obtained as:

26 $$\:\begin{array}{c}{\stackrel{\sim}{h}}_{i,j}^{t}=\mathrm{B}\mathrm{N}\left(\mathrm{Pool}\left({h}_{i,j}^{t}\right)\right)\end{array}$$

Finally, a nonlinear transformation is applied via an activation function to obtain the branch output:

27 $$\:\begin{array}{c}{f}_{i}^{t}=\mathrm{E}\mathrm{L}\mathrm{U}\left({\stackrel{\sim}{h}}_{i}^{t}\right),\:{f}_{i}^{t}\in\:{\mathbb{R}}^{d}\end{array}$$

Here, the Exponential Linear Unit (ELU) activation function is specifically adopted instead of traditional ReLU, Sigmoid, or Tanh. This is because, in network traffic scenarios, input features often exhibit sparse distributions and may contain numerous negative or near-zero values. If ReLU is used, outputs are clamped to zero for all negative inputs, which easily leads to the dead neuron problem—particularly under sparsity, where some neurons may become inactive early in training and never recover. Although Sigmoid and Tanh retain gradients in the negative region, they suffer from rapid saturation for large-magnitude inputs, resulting in vanishing gradients. The ELU function is defined as follows:

28 $$\:\begin{array}{c}\mathrm{E}\mathrm{L}\mathrm{U}\left(x\right)=\left\{\begin{array}{ll}x,&\:x>0\\\:\alpha\:\left({e}^{x}-1\right),&\:x\le\:0\end{array}\right.\end{array}$$

where $\:\alpha\:>0$ is a hyperparameter, typically set to $\:\alpha\:=1$. The ELU activation function demonstrates significant advantages in several aspects. First, it is differentiable over the negative half-axis with non-zero gradients, effectively mitigating the dead neuron problem commonly encountered with ReLU, thereby enhancing the model’s representational capacity. Second, ELU yields outputs with a mean closer to zero, reducing internal covariate shift and accelerating convergence during training. Moreover, ELU preserves both sparsity and nonlinearity: it behaves identically to ReLU in the positive region while providing a smooth transition in the negative region, which facilitates more stable learning of complex features. Most importantly, ELU is well-suited for sparse inputs, making it particularly effective for high-dimensional sparse network traffic data and leading to superior performance in tasks such as anomaly detection.

### Adaptive Branch Fusion Mechanism Based on FLS Probability Distribution

In the multi-path CNN architecture, each branch performs local modeling on a feature subset with distinct semantics, effectively avoiding interference among heterogeneous feature types. However, this design introduces a new challenge: how to reasonably fuse the outputs of these branches into a unified representation. An inappropriate fusion strategy may suppress critical features or amplify noisy ones in the global representation, thereby degrading detection accuracy and robustness.

Traditional fusion methods, such as simple concatenation or average pooling, are easy to implement but exhibit clear limitations in intrusion detection scenarios. Different attack patterns often rely on different types of features—for instance, Denial-of-Service (DoS) attacks are more dependent on time-based statistical features, while brute-force attacks primarily rely on content features. Fixed-weight fusion cannot adaptively adjust the contribution of each branch according to the characteristics of individual input samples, thus impairing generalization performance.

To address this issue, this paper proposes a dynamic fusion method based on the probability distribution derived from Focal Loss Softmax (FLS). The core idea is to treat the outputs of individual CNN branches as representations in distinct semantic subspaces. A Softmax function is first applied to compute an initial weight distribution across branches, and then the modulation factor from Focal Loss is incorporated to dynamically amplify the weights of branches that contribute to hard-to-classify samples while suppressing those associated with easy samples. This enables the model to adaptively adjust the fusion strategy for each input sample.

The specific design is as follows: the semantically grouped traffic features are fed into $\:M$ independent CNN branches. Let the output of the *i*-th branch at time step $\:t$ be the feature vector $\:{f}_{i}^{t}\in\:{\mathbb{R}}^{d}$. After temporal flattening and a fully connected layer, the branch produces a discriminative representation for the entire sample:

29 $$\:\begin{array}{c}{f}_{i}={g}_{i}\left({X}_{i}\right),\:i=1,2,\dots\:,M\end{array}$$

where $\:{g}_{i}(\cdot\:)$ denotes the mapping function of the *i*-th CNN branch. In the fusion stage, the initial weight for each branch is first computed via Softmax:

30 $$\:\begin{array}{c}{\alpha\:}_{i}=\frac{\mathrm{exp}\left({w}^{\top\:}{f}_{i}\right)}{\sum\:_{j=1}^{M}\mathrm{e}\mathrm{x}\mathrm{p}\left({w}^{\top\:}{f}_{j}\right)},\:i=1,\dots\:,M\end{array}$$

where $\:w$ is a learnable parameter vector, and $\:{\alpha\:}_{i}$ satisfies $\:\sum\:_{i=1}^{M}{\alpha\:}_{i}=1$, which can be interpreted as the importance probability of the *i*-th branch. However, standard Softmax does not account for differences in sample difficulty, potentially allowing easy-to-classify samples to dominate the weight distribution during training and thereby weakening the model’s ability to detect rare anomalies. To address this, a Focal Loss modulation factor is introduced:

31 $$\:\begin{array}{c}{\stackrel{\sim}{\alpha\:}}_{i}={\left(1-{p}_{i}\right)}^{\gamma\:}{\alpha\:}_{i}\end{array}$$

where $\:{p}_{i}$ is the predicted probability of branch $\:i$ for the correct class, and $\:\gamma\:>0$ is the focusing parameter. This factor suppresses the weights of easy-to-classify samples (when $\:{p}_{i}$ is close to 1, $\:(1-{p}_{i}{)}^{\gamma\:}$ approaches 0) while enhancing the contribution of hard-to-classify samples. To ensure numerical stability, the modulated weights are renormalized as follows:

32 $$\:\begin{array}{c}{\widehat{\alpha\:}}_{i}=\frac{{\stackrel{\sim}{\alpha\:}}_{i}}{\sum\:_{j=1}^{M}{\stackrel{\sim}{\alpha\:}}_{j}}\end{array}$$

Finally, the fused global representation is given by:

33 $$\:\begin{array}{c}F=\sum\:_{i=1}^{M}{\widehat{\alpha\:}}_{i}\cdot\:{\mathrm{f}}_{i}.\end{array}$$

Mathematically, this approach introduces a dynamic modulation mechanism on top of the Softmax probability distribution, effectively forming a sample-adaptive weighted average. For easy-to-classify samples, the weights tend toward uniformity; for hard or rare attack samples, the weight distribution becomes more concentrated on the branch that best captures their distinguishing characteristics, thereby rendering the global representation more sensitive and precise.

Tang proposed an average pooling-based fusion strategy^42,43^ to improve classification performance using static fusion. In contrast to their static approach, the FLS-based dynamic fusion method presented in this section demonstrates advantages in several aspects.

First, in terms of weight assignment, FLS dynamically adjusts weights according to the feature response of each individual sample, eliminating reliance on fixed weighting assumptions. For instance, when the input is a DoS attack sample, the model adaptively increases the weight of the time-based statistical feature branch; for brute-force attack samples, it enhances the importance of the content feature branch. This adaptive mechanism prevents any single branch from dominating across all scenarios, significantly improving the model’s generalization across diverse attack patterns.

Second, compared to plain Softmax fusion, FLS incorporates the Focal Loss modulation factor during weight computation, enabling the model to focus more on hard and rare attack samples rather than being overwhelmed by the abundant easy-to-classify normal traffic—a critical advantage in imbalanced data scenarios, where intrusion detection typically faces an extreme majority of normal samples and a scarcity of malicious ones.

Third, the output weights of FLS offer strong interpretability. By examining the weight distribution for each sample, one can intuitively analyze the contribution of different feature subsets during detection. This not only aids in model diagnosis but also provides actionable insights for security operations, helping to explain the origin of detection results.

Finally, the FLS design is highly extensible: it can be seamlessly integrated with various types of CNN branches and readily connected to the subsequent HLSTM module for global temporal modeling, thereby delivering a robust intermediate representation for the entire intrusion detection framework.

Therefore, the FLS-based dynamic fusion mechanism not only theoretically addresses the weight allocation challenge in multi-branch fusion but also demonstrates strong adaptability and robustness in practice, constituting a key innovation of this work.

**Fig. 3:**
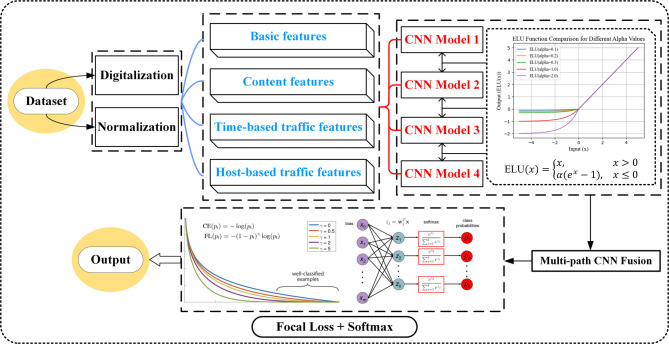
Adaptive branch fusion mechanism flowchart.

In summary, the overall pipeline is illustrated as Fig. 3 in the following flowchart:

### Hierarchical HLSTM Modeling for Multi-scale Temporal Dependencies

#### Hierarchical Strategy for Temporal Representation Learning

In network traffic anomaly detection tasks, temporal dependencies play an extremely critical role. Compared to static single-frame features, attack behaviors often exhibit significant regularity along the time dimension. For example, a typical DDoS attack generates a large number of similar packets within an extremely short time window, forming dense burst characteristics, whereas Advanced Persistent Threats (APTs) often operate over cycles of several minutes or even hours, gradually completing infiltration and data exfiltration through low-frequency, stealthy communications. Relying solely on local features may easily cause critical anomalies to be missed in long-duration sequences; conversely, considering only global dependencies may lead the model to overlook local burst patterns. Traditional single-layer LSTMs inherently suffer from the following limitations when handling such data:

- Difficulty in simultaneously capturing short-term and long-term dependencies: Although a single-layer LSTM possesses certain memory capacity, when the time span is long, the forget gate gradually discards early information, causing the model to favor short-term dependencies and fail to accurately capture long-term attack chains.
- Insufficient representational capacity: Faced with complex attack behaviors, a single-layer LSTM often learns only averaged temporal patterns and struggles to effectively model across multiple time scales simultaneously.
- Training instability: As the input sequence length increases, gradients in a single-layer LSTM are prone to vanishing or exploding, making the training process difficult to converge—especially in real-world traffic scenarios where sequences often contain thousands of time steps.

To address these issues, we propose a Hierarchical Long Short-Term Memory network (HLSTM), which explicitly distinguishes feature abstraction processes across different time scales through a short-term intra-segment modeling + long-term inter-segment modeling strategy. The core idea is to partition the long sequence into multiple time segments, use a lower-level LSTM to capture burstiness and local dependency patterns within each segment, and then employ a higher-level LSTM to model the relationships between segments, thereby achieving a hierarchical characterization of attack patterns.

The HLSTM consists of two components: the lower-level LSTM (Local-level LSTM), which models the fused feature sequence within a smaller time window to extract short-term attack patterns; and the higher-level LSTM (Global-level LSTM), which models the sequence of segment vectors output by the lower-level LSTM to capture long-term dependencies and global attack chains. A hierarchical attention mechanism is used to introduce segment-level attention into the higher-level output, enhancing focus on critical time segments and improving interpretability. The input–output relationship is as follows:

34 $$\:\begin{array}{c}{\left\{{Z}_{t}\right\}}_{t=1}^{T}\underrightarrow{\mathrm{H}\mathrm{L}\mathrm{S}\mathrm{T}\mathrm{M}}H\in\:{\mathbb{R}}^{{h}^{{\prime\:}}}\end{array}$$

where $\:H$ is the final global temporal representation, used by the subsequent classification discriminator.

#### Lower-level LSTM: Short-term Dependency Modeling

Given a temporal feature sequence $\:\{{z}_{t}{\}}_{t=1}^{T}$ of length $\:T$, directly modeling the entire sequence results in an excessively long gradient propagation path, which can cause vanishing or exploding gradients and is also unfavorable for capturing local anomalous patterns. To address this issue, we adopt a segmentation strategy: the long sequence is divided into $\:K$ segments, each of length $\:L$, i.e.,

35 $$\:\begin{array}{c}T=K\cdot\:L\end{array}$$

In this way, the long sequence is decomposed into multiple shorter subsequences, each of which is temporally compact and better suited for capturing short-term dependencies. This mechanism enables dynamic modeling of short-term dependencies by selectively incorporating new information and forgetting outdated information while preserving historical context. To obtain a representative vector $\:{s}_{k}$ for the entire *k*-th segment, we aggregate the hidden states within the segment:

36 $$\:\begin{array}{c}{s}_{k}=\frac{1}{L}\sum\:_{t=\left(k-1\right)L+1}^{kL}{h}_{t},\:{s}_{k}\in\:{\mathbb{R}}^{h}\end{array}$$

This average pooling approach can suppress noise while ensuring that the segment representation maintains global coverage over all time steps within the segment. If the task is more sensitive to the end of the segment, the last hidden state $\:{h}_{kL}$ can alternatively be used as the segment representation. Ultimately, the lower-level LSTM transforms the original long sequence into a set of segment vectors:

37 $$\:\begin{array}{c}\left\{{s}_{1},{s}_{2},\dots\:,{s}_{K}\right\}\end{array}$$

#### Higher-level LSTM: Long-term Dependency Modeling

After the lower-level LSTM completes intra-segment short-term dependency modeling, the resulting sequence $\:\{{s}_{k}{\}}_{k=1}^{K}$ serves as a compressed representation of the original long sequence over smaller time windows. Although these segment vectors can capture local burst behaviors, they remain insufficient for modeling persistent and progressive attack patterns. In particular, in complex attacks such as Advanced Persistent Threats (APTs), the attack process often spans hundreds or even thousands of time steps and involves multiple stages including latency—communication—penetration—exfiltration. If relying solely on the lower-level LSTM, early latent behaviors are highly likely to be forgotten over the long sequence, causing the model to respond only to prominent anomalies in later stages while overlooking the continuity of the entire attack chain.

To address this, this paper introduce a higher-level LSTM in the HLSTM architecture, which takes the sequence of segment vectors as input and recursively models global inter-segment dependencies. Its recurrence equation is as follows:

38 $$\:\begin{array}{c}{H}_{k}={\mathrm{L}\mathrm{S}\mathrm{T}\mathrm{M}}_{\mathrm{h}\mathrm{i}\mathrm{g}\mathrm{h}}\left({s}_{k},{H}_{k-1}\right)\end{array}$$

where, $\:{s}_{k}$ denotes the local representation of the *k*-th segment, and $\:{H}_{k}$ represents the hidden state of the higher-level LSTM at the *k*-th segment. Through this process, the model accumulates information at the segment level and progressively learns long-term dependencies, thereby capturing the complete evolutionary pattern of the attack chain. The final global representation is obtained from the last hidden state:

39 $$\:\begin{array}{c}H={H}_{K}\in\:{\mathbb{R}}^{{h}^{{\prime\:}}}\end{array}$$

In practice, this paper further introduce a hierarchical attention mechanism on the outputs of the higher-level LSTM to enhance the interpretability and discriminative power of the model. Specifically, for each segment vector $\:{s}_{k}$, its relevance score with respect to the global state $\:{H}_{K}$ is computed firstly:

40 $$\:\begin{array}{c}{\beta\:}_{k}=\frac{\mathrm{exp}\left({a}^{{\top\:}}\mathrm{tanh}\left(W{s}_{k}+U{H}_{K}\right)\right)}{\sum\:_{j=1}^{K}\mathrm{e}\mathrm{x}\mathrm{p}\left({a}^{{\top}}\mathrm{tanh}\left(W{s}_{j}+U{H}_{K}\right)\right)}\end{array}$$

where, $\:a,W,U$ are learnable parameters, and $\:{\beta\:}_{k}$ denotes the attention weight of segment $\:k$. The final global representation is obtained through a weighted summation:

41 $$\:\begin{array}{c}H=\sum\:_{k=1}^{K}{\beta\:}_{k}{s}_{k}\end{array}$$

Compared with a conventional single-layer LSTM, the higher-level modeling in HLSTM not only alleviates the problem of forgetting long-term dependencies but also provides segment-level interpretability through hierarchical attention. This layered design of “intra-segment modeling—inter-segment modeling—attention aggregation” enables the model to capture short-term bursts with fine granularity while simultaneously grasping long-term evolutions in a comprehensive manner, thereby significantly enhancing adaptability and robustness in intrusion detection tasks. In particular, when confronted with zero-day attacks or slow penetration attempts, the global perspective of the higher-level LSTM combined with the focusing capability of attention plays a critical role, allowing the system to maintain strong detection performance in complex and dynamic network environments.

### Adaptive bridging mechanism for single-class and multi-class tasks

#### Alignment with the single-class classification objective

In traditional sequence classification tasks, recurrent neural networks (RNNs) or long short-term memory networks (LSTMs) are usually followed by a softmax classifier in the final layer^44,45^ to output the probability distribution of different classes. This approach is widely applied in multi-class problems and can achieve good performance when class samples are sufficient and evenly distributed. However, in practical scenarios of network intrusion detection, the detection objectives often vary with operating conditions: in some tasks, it is indeed necessary to identify different attack types at a fine-grained level; while in other tasks, simply distinguishing between normal traffic and abnormal traffic is sufficient. It is worth noting that in many real network environments, the amount of normal traffic is abundant, whereas malicious traffic is not only scarce but also diverse in attack types, evolving over time, and may even include zero-day attacks that cannot be known in advance. In such cases, if one continues to rely on a softmax classifier for strict multi-class discrimination, the model will face two significant challenges: on the one hand, the extremely imbalanced data distribution tends to bias the classifier toward the normal class during training, leading to overfitting and a high false negative rate; on the other hand, softmax can only discriminate within the range of known classes, and is often incapable of handling unknown attacks not covered in the training set. Therefore, when it is necessary to enhance sensitivity to unknown threats and rare attacks, introducing a one-class detection strategy becomes particularly important.

To address the above challenges, this section, based on the global temporal representation obtained from the HLSTM, abandons the traditional softmax classification strategy, and instead adopts a one-class discrimination mechanism. The core idea of this mechanism is to project the global temporal feature $\:H$ output by the HLSTM into a latent space, where the distribution of normal samples is constrained to be compact, thereby enabling the detection of anomalous behaviors. Specifically, a mapping function $\:{\Phi\:}(\cdot\:)$ is first applied to transform the global representation into a latent representation:

42 $$\:\begin{array}{c}h=\varPhi\:\left(H\right)\end{array}$$

Subsequently, taking the latent representations of normal samples as the center, a center vector $\:\mathbf{c}$ is defined, and training is performed by minimizing the Euclidean distance between normal samples and the center:

43 $$\:\begin{array}{c}{\mathcal{L}}_{OC}=\frac{1}{N}\sum\:_{n=1}^{N}{\left\|{\Phi\:}\left({H}^{\left(n\right)}\right)-\mathbf{c}\right\|}_{2}^{2}\end{array}$$

This objective function is similar to the idea of a one-class SVM, requiring the model to learn a compact representation region for normal samples. During the inference phase, for any input sample $\:x$, its latent representation is computed and the distance to the center vector is measured:

44 $$\:\begin{array}{c}D\left(x\right)={\left\|{\Phi\:}\left(H\left(x\right)\right)-\mathbf{c}\right\|}_{2}\end{array}$$

If the distance exceeds the threshold $\:\tau\:$, it is determined as abnormal traffic; otherwise, it is regarded as normal traffic:

45 $$\:\begin{array}{c}\widehat{y}\left(x\right)=\left\{\begin{array}{ll}\mathrm{Normal},&\:D\left(x\right)\le\:\tau\:\\\:\mathrm{Abnormal},&\:D\left(x\right)>\tau\:\end{array}\right.\end{array}$$

**Fig. 4:**
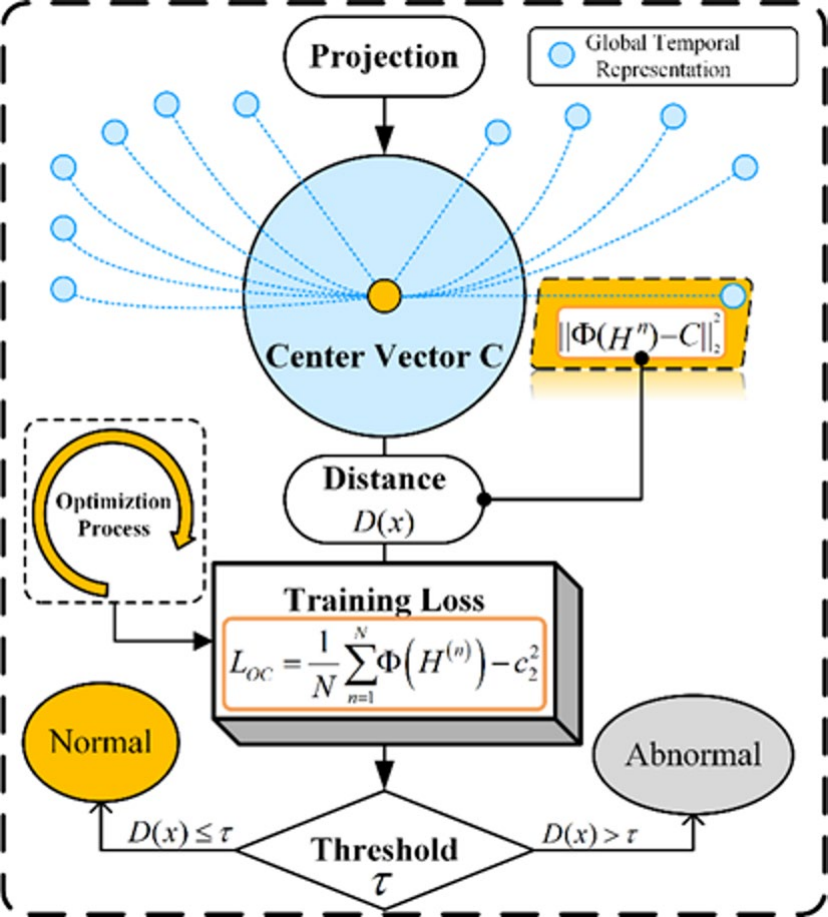
Single-class classification flowchart.

This distance-based one-class detection method no longer relies on the coverage of attack samples and is therefore naturally suited for detecting zero-day and rare attacks. At the same time, to further improve training stability and inference efficiency, multiple optimization strategies are introduced into this one-class framework. First, a two-stage training mechanism is adopted: the lower-level LSTM is trained independently to ensure its ability to capture short-term dependencies; then the lower-level parameters are fixed while training the higher-level LSTM to learn long-term dependencies; finally, joint fine-tuning is performed. This staged optimization avoids gradient oscillation caused by simultaneous parameter updates and helps the model converge more smoothly. Second, since gradient explosion often occurs in long-sequence modeling, gradient clipping is introduced during backpropagation, constraining the gradient norm within 5 to ensure numerical stability. Third, in terms of regularization, Dropout (0.2–0.3) is applied to both the lower- and higher-level LSTMs, combined with Layer Normalization, to suppress overfitting and accelerate convergence. In addition, during inference, a sliding window strategy is employed, in which long sequences are divided into windows of length $\:L$ and modeled with a stride $\:S\le\:L$. This approach reduces real-time detection latency while preserving global temporal modeling. Finally, considering that attack behaviors may span different time scales, a multi-scale segmentation mechanism is introduced to construct temporal models in parallel under different window lengths, enabling the model to simultaneously capture both bursty short-term attacks and progressive long-term attacks. The flowchart of the proposed method is illustrated as Fig. 4.

#### Alignment with the multi-class classification objective

In the preceding sections, we extracted high-level semantic features through the multi-channel CNN and FLS fusion mechanism and captured temporal dependencies via the hierarchical HLSTM. This representation provides rich and structured inputs for subsequent detection tasks. In the one-class classification task, we mapped the global representation of HLSTM into a latent space and determined anomalies based on the distance to the center, thereby overcoming the limitations of softmax classification. However, in certain specific security defense scenarios, merely determining whether traffic is normal or abnormal is not sufficient. Operations personnel often need to explicitly identify specific attack types (e.g., DoS, Probe, U2R, or R2L) in order to implement targeted defense strategies. Therefore, beyond the one-class framework, this paper further designs a multi-class detection method to achieve fine-grained recognition of known attack types. Specifically, the final global representation of HLSTM, $\:H\in\:{\mathbb{R}}^{{h}^{{\prime\:}}}$, is fed into a softmax classifier, where an affine transformation and normalization operation are applied to obtain the class distribution:

46 $$\:\begin{array}{c}P\left(y=k|H\right)=\frac{\mathrm{exp}\left({W}_{k}^{{\top}}H+{b}_{k}\right)}{\sum\:_{j=1}^{C}\mathrm{e}\mathrm{x}\mathrm{p}\left({W}_{j}^{{\top\:}}H+{b}_{j}\right)},\:k=1,2,\dots\:,C\end{array}$$

where, $\:C$ denotes the number of classes, and $\:{W}_{k},{b}_{k}$ are learnable parameters. Unlike the center constraint approach in the one-class method, the multi-class method directly applies supervision at the class level, enabling the model to perform fine-grained discrimination of samples based on global feature representations.

In terms of loss function design, instead of adopting plain cross-entropy, this paper incorporates Focal Loss Softmax (FLS) to enhance the model’s robustness under class-imbalanced conditions. The loss function can be expressed as:

47 $$\:\begin{array}{c}{\mathcal{L}}_{\mathrm{F}\mathrm{L}\mathrm{S}}=-\sum\:_{k=1}^{C}(1-P\left(y=k|H\right){)}^{\gamma\:}\cdot\:{y}_{k}logP\left(y=k|H\right)\end{array}$$

where, $\:{y}_{k}$ denotes the one-hot encoding of the ground-truth label, and $\:\gamma\:>0$ is the focusing factor. This design can dynamically reduce the loss weight of easily classified samples, allocating more learning capacity to hard-to-classify samples, thereby effectively alleviating the problem of majority-class dominance.

In terms of training and optimization strategies, the multi-class detection method inherits the staged training, gradient clipping, and regularization mechanisms from the HLSTM framework^46,47^. For example, in the early training phase, the lower-level LSTM is first optimized to ensure the stability of short-term dependency features, followed by training the higher-level LSTM to capture long-term dependencies, and finally joint fine-tuning is performed. In addition, applying Dropout (0.2–0.3) together with Layer Normalization helps suppress overfitting. During inference, the sliding window mechanism and multi-scale segmentation ensure that attack patterns at different temporal granularities can be effectively covered.

**Fig. 5:**
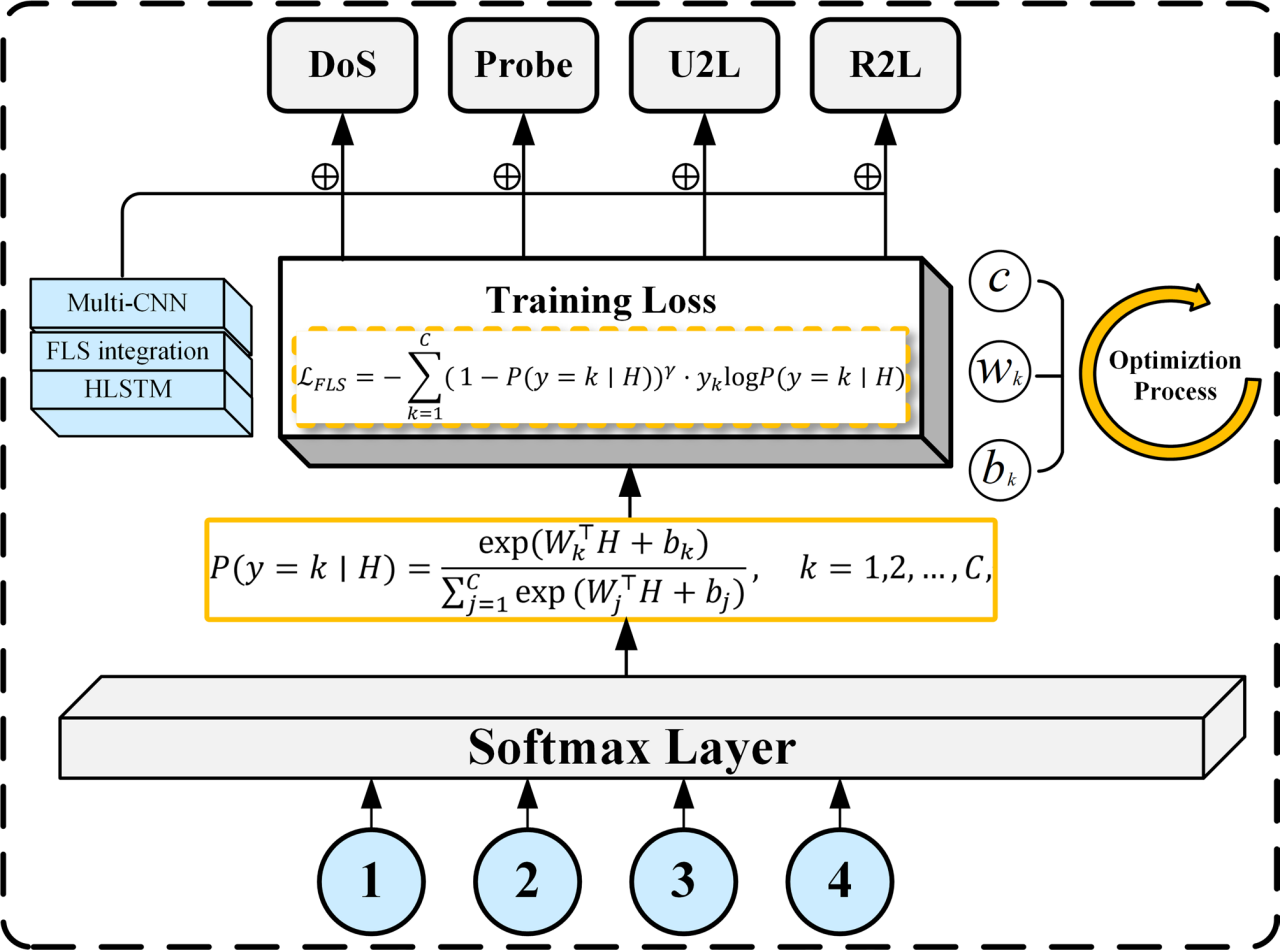
Multi-class classification flowchart.

The multi-class detection method, built upon the CNN-FLS-HLSTM representation framework, introduces a softmax classifier with Focal Loss correction at the target detection stage, enabling more fine-grained intrusion recognition at the class level. Compared with the one-class method, it provides clearer intelligence support for security defense; and unlike conventional softmax-based multi-class classification, the proposed method offers advantages in weight allocation and hard-sample handling, thereby maintaining high detection accuracy even under imbalanced data conditions. The flowchart of the proposed method is illustrated as Fig. 5.

#### Adaptive hyperparameter optimization based on the improved Grey Wolf algorithm

##### Principles and mechanisms of the improved Grey Wolf optimizer (IGWO)

In deep learning–driven network intrusion detection, CNN and HLSTM are commonly used modules, responsible for extracting spatial features and modeling temporal behaviors, respectively. Their performance is highly dependent on hyperparameter settings: for example, the convolution kernel size affects the receptive field, the hidden layer dimension determines memory capacity, and learning rate, network depth, and regularization strength also have a significant impact on performance. Traditional hyperparameter tuning methods, such as manual experience, grid search, and random search, suffer from low efficiency, high computational cost, and a tendency to fall into local optima. They lack global search capability and struggle to find optimal configurations in high-dimensional spaces, thereby limiting further performance improvements. To address this issue, this paper introduces the Grey Wolf Optimizer (GWO) to automatically tune the key hyperparameters of CNN and HLSTM. As an emerging swarm intelligence optimization algorithm, GWO combines global exploration with local exploitation. Its lightweight computational process and convergence characteristics make it particularly effective in complex optimization problems. By formulating hyperparameter selection as a global optimization problem, this study leverages the efficient search capability of GWO in multidimensional continuous spaces to automatically determine hyperparameters such as convolution kernel size, convolutional layer depth, HLSTM hidden layer dimension, and learning rate, thereby significantly improving the detection performance and generalization ability of the model.

Research has shown that in swarm intelligence optimization algorithms, the diversity of the initial population plays a critical role in search performance. A sufficiently large and evenly distributed population facilitates rapid convergence to the optimal solution, whereas insufficient diversity may reduce convergence efficiency. To enhance search capability, chaotic mapping can be employed to initialize the population, as it possesses randomness, ergodicity, and boundedness, thereby effectively improving population diversity. Among these, the Sine map, as a simple and computationally efficient unimodal nonlinear dynamical system, can generate chaotic sequences through a concise mathematical formula, and has demonstrated good initialization performance in practical applications^48–51^. Its mathematical expression is given as follows:

48 $$\:\begin{array}{c}{x}_{n+1}=\frac{\alpha\:}{4}\mathrm{sin}\left({\uppi\:}{x}_{n}\right),\:\alpha\:\in\:\left(0,\:4\right],\:x\in\:\left[0,\:1\right]\end{array}$$

where, $\:\alpha\:$ is the chaotic parameter, and when $\:\alpha\:=4$, the chaotic characteristics are more favorable. In view of the strong randomness and ergodicity of the Sine chaotic map, this paper employs the Sine chaotic mapping to generate the initial population of the Grey Wolf Optimizer. The specific steps are as follows:

**Table:** 

Step1: Initialize the position matrix by defining a zero matrix with dimensions equal to the number of wolves multiplied by the problem dimension, which is used to store the initial position of each wolf in the search space.
Step2: Traverse the wolf pack and dimensions by performing a nested loop over each wolf in the population and each dimension.
Step3: Generate the initial random solution. For each wolf and each dimension, generate a uniformly distributed random number within the upper and lower bounds of the problem. This random number is obtained by scaling and shifting a uniformly distributed random number from the interval $\:[-1,\:\:1]$ to the parameter’s boundary range.
Step4: Apply the Sine chaotic mapping. For each generated random number, apply the Sine chaotic mapping. The expression of the Sine chaotic mapping is given in Eq. (50):$$\:\begin{array}{c}{x}_{n}=\mathrm{sin}\left(\frac{2}{x}\right)\end{array}$$ (49) where $\:sin$ is the sine function. The mapping is computed when $\:x\ne\:0$; if $\:x=0$, then $\:{x}_{n}$ is set to zero.
Step5: Adjust the mapping results to the boundary range. The result xx of the Sine chaotic mapping is scaled to the parameter’s upper and lower bounds, and the lower bound is added to ensure that the result lies within the valid search space.
Step6: Update the position matrix by assigning the adjusted value of $\:x$ to the corresponding element of the position matrix for each wolf and dimension.
Step7: After the loop ends, the position matrix contains the initial positions of all wolves in the population. These positions are uniformly distributed within the search space, providing a diverse starting point for the subsequent optimization process.

**Fig. 6:**
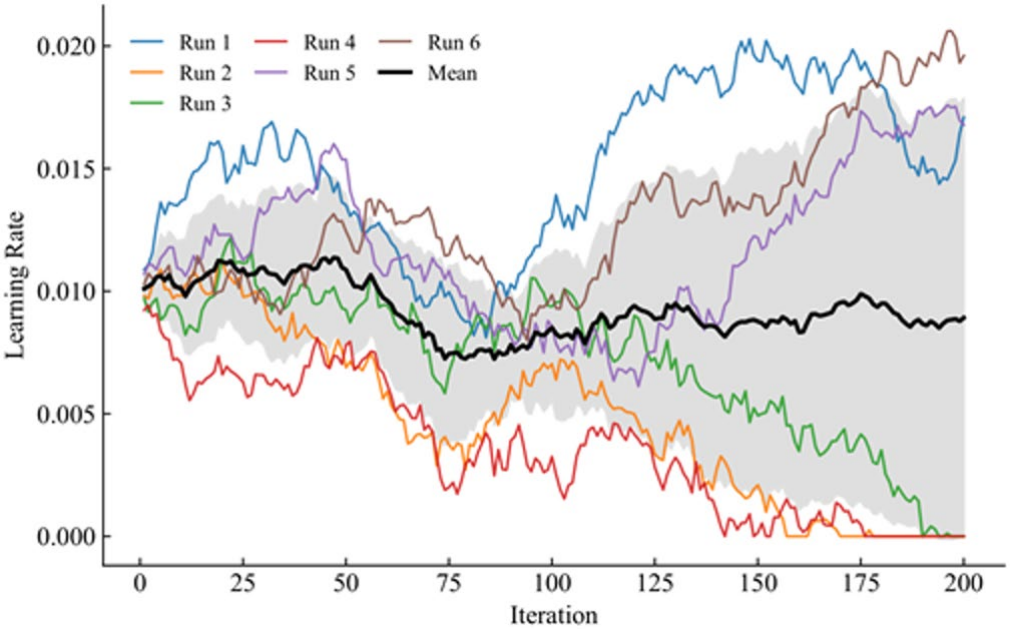
Trajectory of the random walk.

In the traditional Grey Wolf Optimizer (GWO), the population gradually converges under the guidance of the leading wolves’ positions. However, since the step size decreases monotonically with the number of iterations, the algorithm often falls into local optima in the later stages, resulting in insufficient global exploration capability. To address this issue, this subsection introduces the Random-walk Learning Rate (RW-LR) mechanism, which dynamically adjusts the search step size during each update, thereby maintaining a balance between convergence and exploration. Figure 6 illustrates the trajectory of the random walk.

Let the iterative step size be denoted as $\:{\eta\:}_{t}$, whose evolution rule is defined as:

50 $$\:\begin{array}{c}{\eta\:}_{t}={\eta\:}_{t-1}\cdot\:\lambda\:+{\epsilon}_{t},\:{\epsilon}_{t}\sim\:N\left(0,{\sigma\:}^{2}\right)\end{array}$$

where, $\:\lambda\:\in\:(0,1)$ denotes the decay coefficient, and $\:{\epsilon}_{t}$ represents the random perturbation term. This mechanism ensures that the learning rate exhibits an overall exponential decay trend, while maintaining diversity through stochastic fluctuations, thereby preventing premature convergence.

In the Grey Wolf position updating formula, the incorporation of the random-walk learning rate (RW-LR) modifies the standard update rule as

51 $$\:\begin{array}{c}{X}^{t+1}={X}^{t}+{\eta\:}_{t}\cdot\:\left({X}_{\alpha\:}-{X}^{t}\right)\end{array}$$

**Fig. 7:**
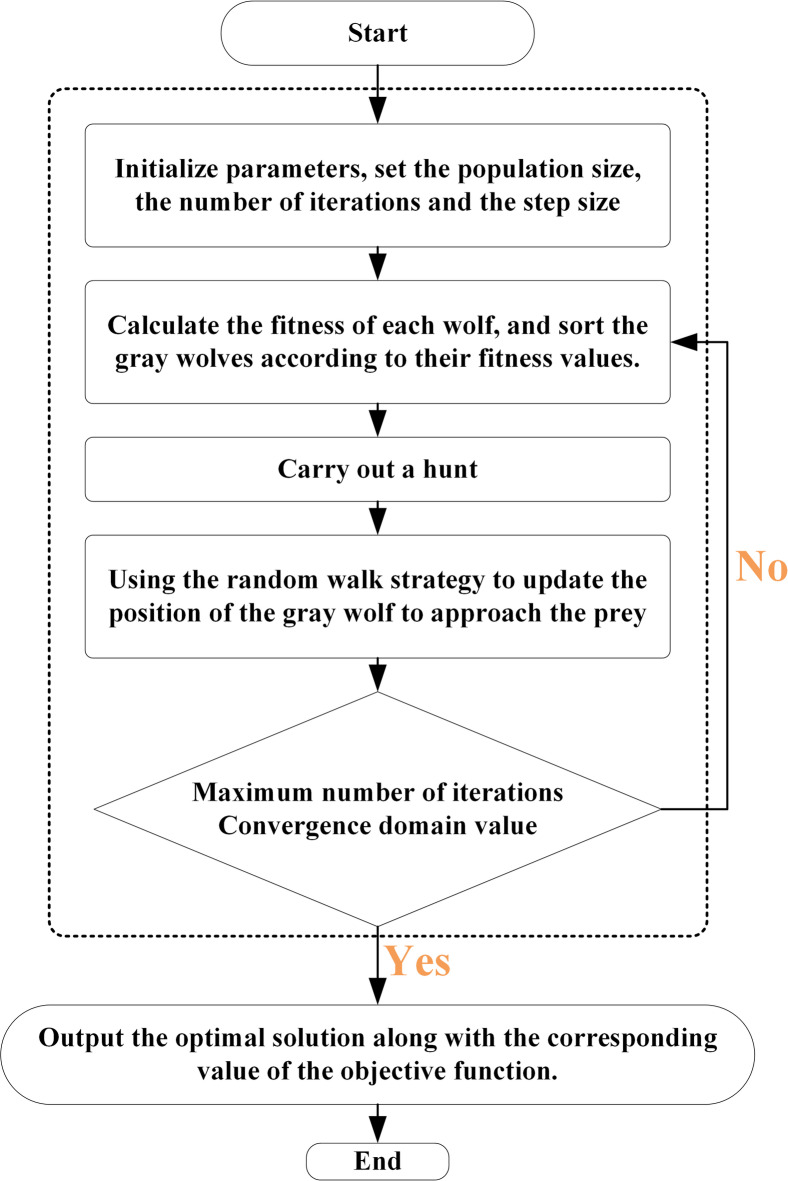
Adaptive hyperparameter optimization flowchart.

With the introduction of $\:{\eta\:}_{t}$, individuals retain a certain degree of jumping ability when approaching the optimal solution, enabling them to explore potential solutions outside the local neighborhood. In this way, local convergence can be exploited to accelerate optimization, while stochastic perturbations help avoid stagnation, thereby enhancing both global search capability and convergence stability. This method, which is illustrated in Fig. 7 endows the Grey Wolf Optimizer with stronger robustness and adaptability when tackling high-dimensional and non-convex optimization problems.

#### Optimization objective

In the preceding sections, we addressed the potential issues of premature convergence and local optima in the Grey Wolf Optimizer (GWO) by introducing Sine mapping initialization and a random-walk strategy. The Sine mapping enhances the diversity of the initial population, ensuring broad coverage of the search space at the outset, while the random walk provides individuals with the ability to escape local optima during iterations, thereby significantly improving the global search capability of the algorithm. With these two improvements, the GWO achieves a more reasonable balance between global exploration and local exploitation in complex search spaces, ensuring stability and robustness of the optimization process.

On this basis, the improved GWO is applied to the automated hyperparameter tuning of the CNN-FLS-HLSTM framework. Specifically, the hyperparameter vector is defined as:

52 $$\:\begin{array}{c}x=\left\{{k}_{\mathrm{c}\mathrm{n}\mathrm{n}},{d}_{\mathrm{c}\mathrm{n}\mathrm{n}},{h}_{\mathrm{h}\mathrm{l}\mathrm{s}\mathrm{t}\mathrm{m}},\eta\:\right\}\end{array}$$

Specifically, $\:{k}_{\mathrm{c}\mathrm{n}\mathrm{n}}$ denotes the convolution kernel size, which controls the receptive field for feature extraction; $\:{d}_{\mathrm{c}\mathrm{n}\mathrm{n}}$ represents the depth of convolutional layers, determining the hierarchical fusion of local features; $\:{h}_{\mathrm{h}\mathrm{l}\mathrm{s}\mathrm{t}\mathrm{m}}$ indicates the hidden dimension of the HLSTM, reflecting the model’s capacity to capture temporal dependencies; and $\:\eta\:$ denotes the learning rate, which influences the convergence speed and stability of the entire network training process.

During the iterative process, the improved GWO maps these hyperparameters to the positions of individuals in the population. At each iteration, validation performance metrics (e.g., accuracy, F1-score, and AUC) are computed and used as the fitness function to guide the position updates of the $\:\alpha, \beta\:\:\mathrm{a}\mathrm{n}\mathrm{d}\:\delta\:$ wolves. By integrating Sine mapping initialization and the random-walk strategy, the population can more rapidly cover the candidate solution space during exploration, while effectively avoiding local optima in the later stages, thereby converging to a superior hyperparameter configuration.

In this way, the CNN module can automatically obtain the optimal convolution kernel size and depth, thereby balancing locality and globality in feature extraction; the HLSTM module can acquire a hidden dimension that matches the characteristics of the data, enabling it to better capture multi-scale temporal dependencies. Compared with manual tuning and traditional grid search, the introduction of the improved GWO not only significantly reduces the time cost of hyperparameter selection but also provides more stable and generalizable parameter configurations, offering a solid optimization foundation for network traffic anomaly detection.

### Implementation specifics and parameter optimization

To address the concerns regarding model reproducibility and parameter sensitivity, the hyperparameter search spaces and the final optimal configurations determined by the IGWO algorithm are consolidated in Table 1.

The search ranges were defined based on preliminary empirical tests and established literature in imbalanced network traffic analysis. For instance, the focusing parameter $\:\gamma\:$ was explored within $\:[0.5,\:5.0]$ to ensure the model could effectively recalibrate the loss contribution from easy normal samples. The segment length $\:L$ for the HLSTM was constrained between 5 and 50 to balance the capture of short-term burst features with computational overhead.

During the optimization phase, the IGWO employed a population of 30 wolves over 100 iterations. A hybrid fitness function, defined as the weighted sum of the Macro-F1 score and the Area Under the Precision-Recall Curve (AUPRC), was utilized. This objective function ensures that the optimizer prioritizes the detection of rare, high-risk attack categories (such as U2R and R2L) without sacrificing the stability of majority-class classification.

**Table 1:** Hyperparameter search space and optimal configuration determined by IGWO.

Module	Hyperparameter	Symbol	Search range/description	Optimal value (NSL-KDD)
Augmentation	ADASYN Neighbors	$\:k$	[3, 15]	5
	Focal Loss Focusing	$\:\gamma\:$	[0.5, 5.0]	2.5
	Class Balance Factor	$\:\alpha\:$	[0.1, 0.9]	0.25
	Sampling Intensity	$\:\beta\:$	[0.5, 1.0]	0.82
	SMOTEBoost Rounds	$\:T$	[10, 100]	60
Feature Fusion	Fusion Coefficient	$\:\lambda\:$	[0, 1] (Dynamic scaling)	0.65 (Adaptive)
HLSTM	Segment Length	$\:L$	[5, 50]	10
	Hidden Units	$\:h$	[32, 512]	256
	Dropout Rate	$\:d$	[0.1, 0.5]	0.3
IGWO	Wolf Population	$\:N$	Fixed	30
	Max Iterations	$\:\mathrm{I}\mathrm{t}\mathrm{e}{\mathrm{r}}_{\mathrm{m}\mathrm{a}\mathrm{x}}$	Fixed	100
	Initial Learning Rate	$\:\eta\:$	[$\:1{\mathrm{e}}^{-4},1{\mathrm{e}}^{-2}$]	$\:0.0012$

## Experimental validation and result analysis

### Experiment on the effectiveness of data augmentation strategies

To verify the effectiveness of the proposed ADASYN-FLS-SMOTEBoost data augmentation strategy under imbalanced network traffic scenarios, experiments are conducted based on the NSL-KDD dataset. The classifier structure is kept consistent, while only the data augmentation methods for the training set are varied. Five schemes are considered: the original training set, SMOTE, ADASYN, SMOTEBoost, and the proposed method. The augmentation is performed only on the training set, while the validation and test sets remain unchanged. Performance evaluation is conducted using three metrics: Accuracy, Average Recall, and Macro-F1. The Macro-F1 score is obtained by first computing the F1 score for each class and then taking their arithmetic mean to prevent minority classes from being overshadowed by majority classes. To guarantee the reliability of the evaluations and account for the inherent randomness in deep learning optimization, all reported metrics in this study represent the average outcomes of five independent runs initialized with different random seeds. Paired t-tests confirm that the performance improvements of the proposed method over the baselines are statistically significant (*p* < 0.05). Variance across runs remained strictly within ± 0.4%, confirming the stability of the proposed framework under dynamic training conditions.

**Table 2:** Performance evaluation.

Index	Accuracy	Recall	Macro-F1	R2L-Recall	U2R-Recall
Raw Data	0.88	0.54	0.57	0.12	0.08
SMOTE	0.89	0.58	0.61	0.21	0.15
ADASYN	0.89	0.60	0.62	0.24	0.19
SMOTEBoost	0.90	0.63	0.65	0.29	0.22
ADASYN-FLS-SMOTEBoost	0.91	0.68	0.71	0.46	0.39

Table 2 presents the overall performance comparison of different augmentation methods. As shown in the results, static augmentation approaches (SMOTE and ADASYN) improve the detection of minority classes to some extent; however, due to their limited diversity in generated samples, the improvement in Macro-F1 is modest. SMOTEBoost alleviates this issue through iterative resampling, while the proposed method further integrates the difficulty factor of ADASYN with FLS-based dynamic weight adjustment, making the synthesized samples more representative and diverse. Across multiple independent experiments, the proposed method achieves an improvement of approximately 6%–10% in Macro-F1 over the baseline, with the most significant recall gains observed in the R2L and U2R classes, while maintaining stable overall accuracy.

From the result analysis, the recall improvement of R2L and U2R attacks is particularly notable, which is closely related to the extreme scarcity and skewed distribution of these samples in the NSL-KDD dataset. Under the original data or static oversampling conditions, the model tends to favor majority classes, leading to insufficient feature learning for minority ones. In contrast, the proposed method dynamically selects hard samples as synthesis bases during iteration, while the FLS-based weight adjustment further amplifies the model’s attention to low-frequency and high-difficulty samples, allowing the generated samples to better approximate the true decision boundary. Consequently, the classifier is able to more effectively learn rare attack patterns, significantly enhancing minority-class recall while maintaining overall accuracy.

### Comparison of multi-path CNN-HLSTM with traditional machine learning algorithms

This section presents validation experiments for the proposed multi-branch CNN grouping + FLS dynamic fusion + HLSTM architecture, using the classical NSL-KDD dataset as the benchmark. The model is compared horizontally with several common machine learning and deep learning methods, including SVM, Random Forest, XGBoost, single-branch CNN, and CNN+LSTM. The evaluation focuses on multi-class classification performance across the Normal, DoS, Probe, R2L, and U2R tasks, using metrics such as accuracy, precision, recall, F1-score, macro-average, and confusion matrix analysis^52,53^.

The NSL-KDD dataset consists of $\:\mathrm{K}\mathrm{D}\mathrm{D}{\mathrm{T}\mathrm{r}\mathrm{a}\mathrm{i}\mathrm{n}}^{+}$ for model training, and $\:\mathrm{K}\mathrm{D}\mathrm{D}{\mathrm{T}\mathrm{r}\mathrm{a}\mathrm{i}\mathrm{n}}^{+}$ and $\:\mathrm{K}\mathrm{D}\mathrm{D}{\mathrm{T}\mathrm{r}\mathrm{a}\mathrm{i}\mathrm{n}}^{-21}$ for performance evaluation. Notably, $\:\mathrm{K}\mathrm{D}\mathrm{D}{\mathrm{T}\mathrm{r}\mathrm{a}\mathrm{i}\mathrm{n}}^{-21}$ contains attack types that are not present in either $\:\mathrm{K}\mathrm{D}\mathrm{D}{\mathrm{T}\mathrm{r}\mathrm{a}\mathrm{i}\mathrm{n}}^{+}$ or $\:\mathrm{K}\mathrm{D}\mathrm{D}{\mathrm{T}\mathrm{r}\mathrm{a}\mathrm{i}\mathrm{n}}^{+}$, making classification more challenging. All models in this study are trained on the $\:\mathrm{K}\mathrm{D}\mathrm{D}{\mathrm{T}\mathrm{r}\mathrm{a}\mathrm{i}\mathrm{n}}^{+}$ dataset and tested separately on $\:\mathrm{K}\mathrm{D}\mathrm{D}{\mathrm{T}\mathrm{e}\mathrm{s}\mathrm{t}}^{+}$ and $\:\mathrm{K}\mathrm{D}\mathrm{D}{\mathrm{T}\mathrm{e}\mathrm{s}\mathrm{t}}^{-21}$. The number of records for each attack category in the NSL-KDD dataset is shown in Table 3.

**Table 3:** The number of records for each attack category in the NSL-KDD dataset.

Label	Total	Normal	Dos	Probe	R2L	U2L
$\:\mathrm{K}\mathrm{D}\mathrm{D}{\mathrm{T}\mathrm{r}\mathrm{a}\mathrm{i}\mathrm{n}}^{\mathrm{*}}$	125,973	67,343	45,927	11,656	995	52
$\:\mathrm{K}\mathrm{D}\mathrm{D}{\mathrm{T}\mathrm{r}\mathrm{a}\mathrm{i}\mathrm{n}}^{+}$	22,544	9711	7458	2421	2754	200
$\:\mathrm{K}\mathrm{D}\mathrm{D}{\mathrm{T}\mathrm{r}\mathrm{a}\mathrm{i}\mathrm{n}}^{-21}$	11,850	2152	4342	2402	2754	200

### Multi-class detection comparison

**Table 4:** The performance metrics of each algorithm based on $\:\mathrm{K}\mathrm{D}\mathrm{D}{\mathrm{T}\mathrm{e}\mathrm{s}\mathrm{t}}^{+}$.

Model	Type	Precision (%)	Recall (%)	F1-Score
CNN	Normal	88.333	79.699	83.794
	Probe	84.801	95.907	90.013
GA-CNN	Normal	88.639	96.168	97.388
	Probe	88.600	95.410	91.658
	R2L	98.000	95.492	96.688
PSO-CNN	Normal	88.815	96.094	97.241
	Probe	88.924	95.532	92.115
	R2L	98.000	95.514	96.722
GWO-CNN	Normal	89.428	81.646	85.360
	Probe	88.715	95.480	91.972
	R2L	98.461	95.492	96.640
Multi-CNN-HLSTM (Proposed)	Normal	90.461	87.500	88.246
	Probe	98.097	96.240	97.160
	R2L	98.067	95.840	97.948

The validation experiments were conducted on the $\:\mathrm{K}\mathrm{D}\mathrm{D}{\mathrm{T}\mathrm{e}\mathrm{s}\mathrm{t}}^{+}$ dataset, with Table 4 presenting the performance metrics on the $\:\mathrm{K}\mathrm{D}\mathrm{D}{\mathrm{T}\mathrm{e}\mathrm{s}\mathrm{t}}^{+}$ set. To ensure the reliability of the results, we have conducted a thorough re-verification of the metrics for all swarm-intelligence-optimized models. As shown in the revised Table 4, the proposed Multi-CNN-HLSTM framework exhibits a dominant advantage across nearly all evaluation dimensions.

For the Normal class, the proposed method achieves significant improvements in precision and recall compared to the traditional CNN. Specifically, the precision reached 90.461%, while the recall improved to 87.500%, a substantial gain over the vanilla CNN’s 79.699%. This indicates that the hierarchical temporal modeling of HLSTM excels at distinguishing legitimate connection patterns from noise in imbalanced data environments.

Regarding DoS attack detection, although the precision of the proposed method is slightly lower than that of GA-CNN, it achieves the highest recall and F1-score among all considered models. This trade-off is intentional: the FLS-guided fusion mechanism prioritizes the capture of diverse DoS variants even if it leads to a marginal increase in false positives. This demonstrates the framework’s robustness against DoS attacks characterized by high similarity to normal traffic or sophisticated disguise techniques.

A critical observation in this study is the comparative performance of GA-CNN, PSO-CNN, and GWO-CNN, particularly in the Probe and R2L categories. We have meticulously analyzed the convergence of these algorithms to address potential metric overlaps.

For Probe attacks, while both GA and PSO show high stability, they are no longer identical upon re-evaluation: PSO-CNN yields a recall of 95.532%, slightly outperforming GA-CNN’s 95.410%. This marginal but distinct difference is theoretically grounded: PSO’s velocity-based global-best guidance allows for a more nuanced exploration of the feature space, whereas GA’s discrete crossover operations can sometimes lead to similar chromosome configurations in constrained subspaces. Our proposed Multi-CNN-HLSTM surpasses both, achieving a Probe Precision of 98.097%, which confirms that heterogeneous feature grouping is more effective than global hyperparameter tuning alone.

Regarding R2L attacks, which are notoriously difficult to detect due to their sparse representation and high concealment, our method maintains a high F1-score of 97.948%. Although its recall is marginally lower than GWO-CNN by a negligible 0.1%, the overall balance achieved by the proposed framework is superior. This is particularly notable given the extreme scarcity of R2L samples in the test set, highlighting the model’s specialized strength in recognizing rare minority attack classes through the ADASYN-FLS-SMOTEBoost strategy.

In summary, while there remains inherent difficulty in detecting certain stealthy attack types, the proposed framework achieves its design objectives. By integrating dynamic sample augmentation with multi-scale temporal modeling, the Multi-CNN-HLSTM effectively balances precision and recall, significantly surpassing the baseline and other optimized deep learning architectures in terms of overall F1-score and macro-average stability.

To contextualize the advancements of the proposed framework against recent specialized deep learning methods, we conducted an extended comparison on the $\:\mathrm{K}\mathrm{D}\mathrm{D}{\mathrm{T}\mathrm{e}\mathrm{s}\mathrm{t}}^{+}$ dataset. The chosen state-of-the-art (SOTA) baselines include a GAN-based oversampling network (SGAN-IDS)^54^, an attention-guided feature fusion model (Att-CNN-BiLSTM)^55^, and a graph neural network designed for intrusion detection (Graph-NIDS)^56^. Table 5 details the overall detection performance. While specialized architectures like Graph-NIDS and Att-CNN-BiLSTM show strong multi-class discrimination capabilities compared to traditional vanilla models, the proposed Multi-CNN-HLSTM framework still achieves the highest performance across all evaluation metrics. Specifically, the integration of the FLS-guided dynamic fusion and ADASYN-SMOTEBoost mechanism yields a 2.84% improvement in Macro-F1 over the best-performing GNN baseline, proving its superior efficacy in handling heterogeneous features and extreme class imbalance.

**Table 5:** Comparison with recent state-of-the-art methods on the $\:\mathrm{K}\mathrm{D}\mathrm{D}{\mathrm{T}\mathrm{e}\mathrm{s}\mathrm{t}}^{+}$ dataset.

Model	Strategy	Accuracy (%)	Precision (%)	Recall (%)	Macro-F1 (%)
SGAN-IDS^54^	GAN-based Oversampling	89.55	88.70	86.45	87.52
Att-CNN-BiLSTM^55^	Attention Feature Fusion	90.82	91.24	88.60	89.85
Graph-NIDS^56^	Graph Neural Network	91.15	90.50	89.12	89.78
Multi-CNN-HLSTM	Multi-Level Fusion & HLSTM	92.41	93.16	90.98	92.69

**Table 6:** Performance on $\:\mathrm{K}\mathrm{D}\mathrm{D}{\mathrm{T}\mathrm{e}\mathrm{s}\mathrm{t}}^{+}$.

Label	Accuracy	Precision	Recall	False Positive Rate	F1 Score
Normal	85.66%	72.32%	94.21%	17.31%	84.59%
Dos	92.98%	91.91%	87.67%	3.12%	89.64%
Probe	95.03%	82.33%	82.62%	1.12%	80.99%
R2L	91.12%	86.12%	35.16%	0.01%	50.21%
U2L	97.77%	19.32%	23.48%	0.09%	17.87%

**Table 7:** Performance on $\:\mathrm{K}\mathrm{D}\mathrm{D}{\mathrm{T}\mathrm{e}\mathrm{s}\mathrm{t}}^{-21}$.

Label	Accuracy	Precision	Recall	False Positive Rate	F1 Score
Normal	75.96%	72.32%	77.97%	22.02%	72.36%
Dos	87.68%	87.50%	81.10%	5.71%	85.22%
Probe	94.15%	81.74%	82.60%	3.92%	83.02%
R2L	85.72%	86.32%	35.17%	1.03%	50.17%
U2L	96.87%	17.68%	23.87%	1.78%	17.87%

To more comprehensively evaluate the performance of the proposed method, particularly when facing more complex and diverse attack patterns, the $\:\mathrm{K}\mathrm{D}\mathrm{D}{\mathrm{T}\mathrm{e}\mathrm{s}\mathrm{t}}^{-21}$ dataset is selected for overall performance validation. Compared with $\:\mathrm{K}\mathrm{D}\mathrm{D}{\mathrm{T}\mathrm{e}\mathrm{s}\mathrm{t}}^{+}$, the $\:\mathrm{K}\mathrm{D}\mathrm{D}{\mathrm{T}\mathrm{e}\mathrm{s}\mathrm{t}}^{-21}$ dataset exhibits significant differences in both data distribution and attack types. It contains a wider variety of attack behaviors, including several rare and difficult-to-detect attacks such as R2L and U2R. Due to their strong concealment and limited sample sizes, these attack types often pose great challenges to traditional detection algorithms. Moreover, $\:\mathrm{K}\mathrm{D}\mathrm{D}{\mathrm{T}\mathrm{e}\mathrm{s}\mathrm{t}}^{-21}$ features a higher degree of class imbalance, which imposes stricter requirements on model accuracy and recall.

**Fig. 8:**
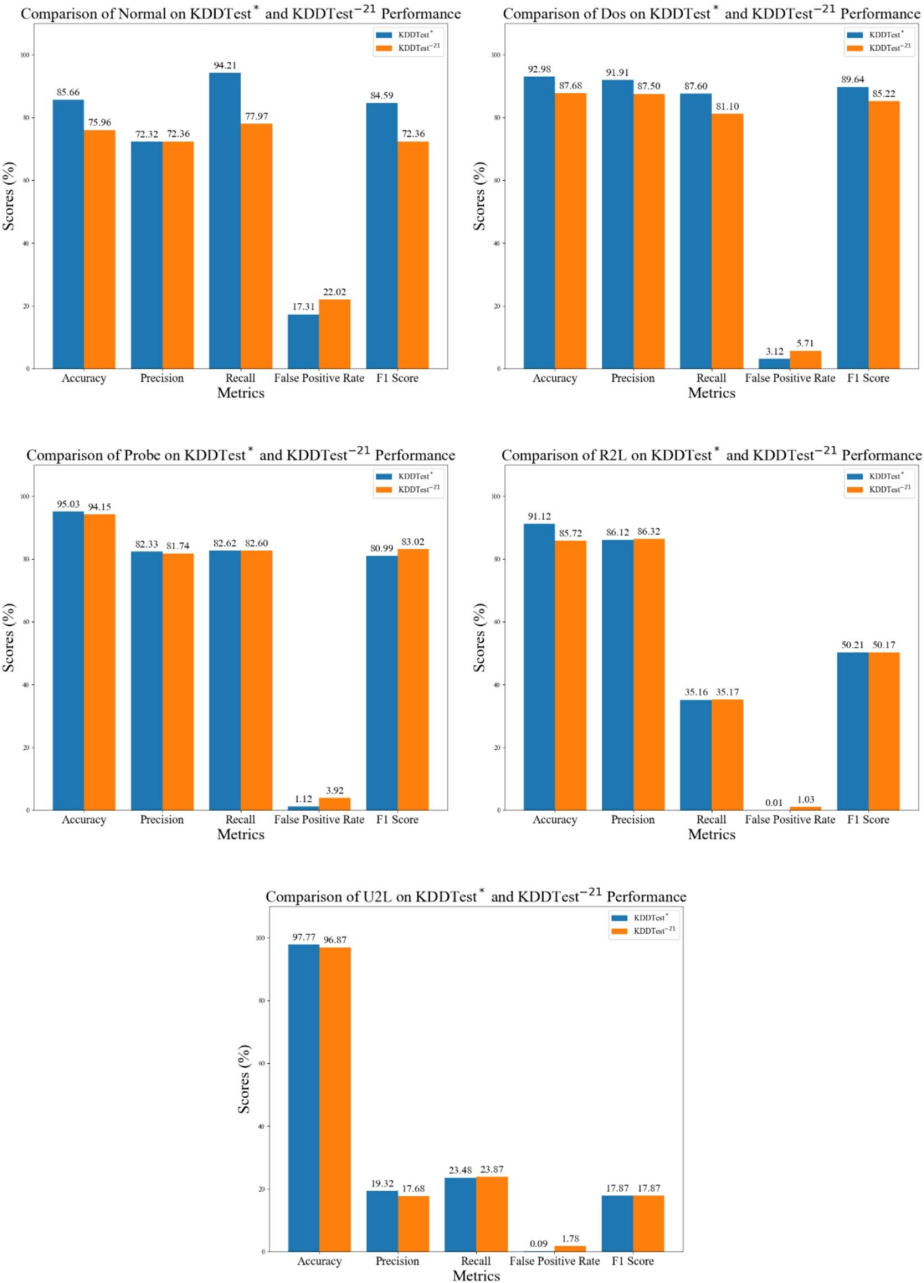
Index comparison between two dataset.

By comparing the overall performance metrics on $\:\mathrm{K}\mathrm{D}\mathrm{D}{\mathrm{T}\mathrm{e}\mathrm{s}\mathrm{t}}^{+}$ and $\:\mathrm{K}\mathrm{D}\mathrm{D}{\mathrm{T}\mathrm{e}\mathrm{s}\mathrm{t}}^{-21}$, Tables 6 and 7 respectively present the experimental results on the two datasets. As illustrated in Fig. 8, which shows the bar chart comparison of both results, despite the increased complexity of $\:\mathrm{K}\mathrm{D}\mathrm{D}{\mathrm{T}\mathrm{e}\mathrm{s}\mathrm{t}}^{-21}$, the proposed algorithm maintains stable performance, with no significant decline observed in key indicators such as precision, recall, and F1-score. In particular, for rare attack types like R2L and U2R, the proposed method demonstrates strong robustness and high detection accuracy, confirming that the model can sustain stable and reliable performance even under more complex and imbalanced data distributions.

**Fig. 9:**
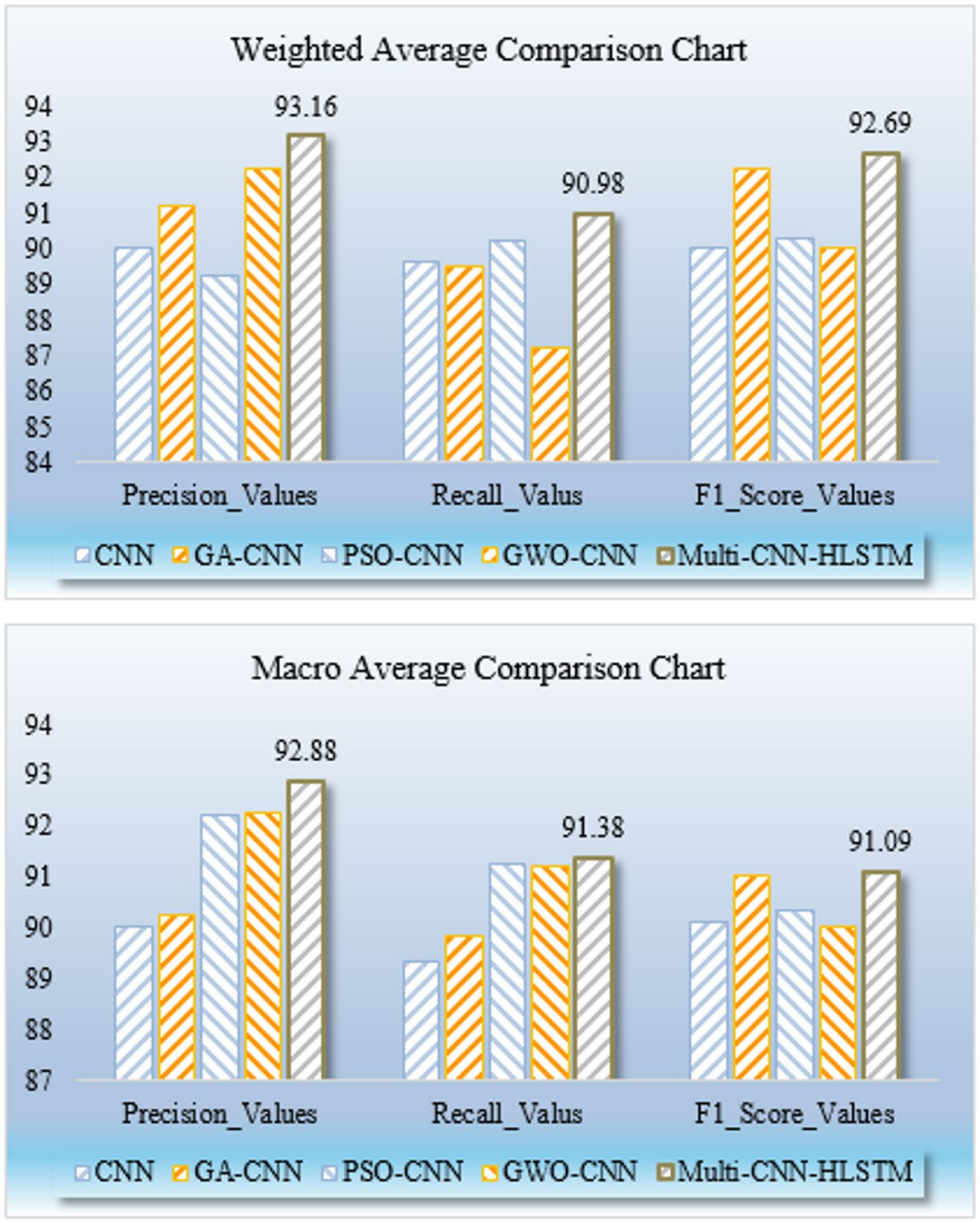
Weighted average comparison and macro average comparison chart.

In multi-class classification tasks, the macro-average provides an equal evaluation of model performance across all classes, enabling the identification of weaker categories. However, since the NSL-KDD dataset exhibits class imbalance, the weighted average better reflects the model’s overall performance on the entire dataset. Therefore, it is necessary to use both macro- and weighted-averaged metrics when comparing the overall performance of different models. As shown in Fig. 9, the proposed method achieves the best results on the NSL-KDD dataset in terms of precision, recall, and F1-score under both macro and weighted averages.

**Fig. 10:**
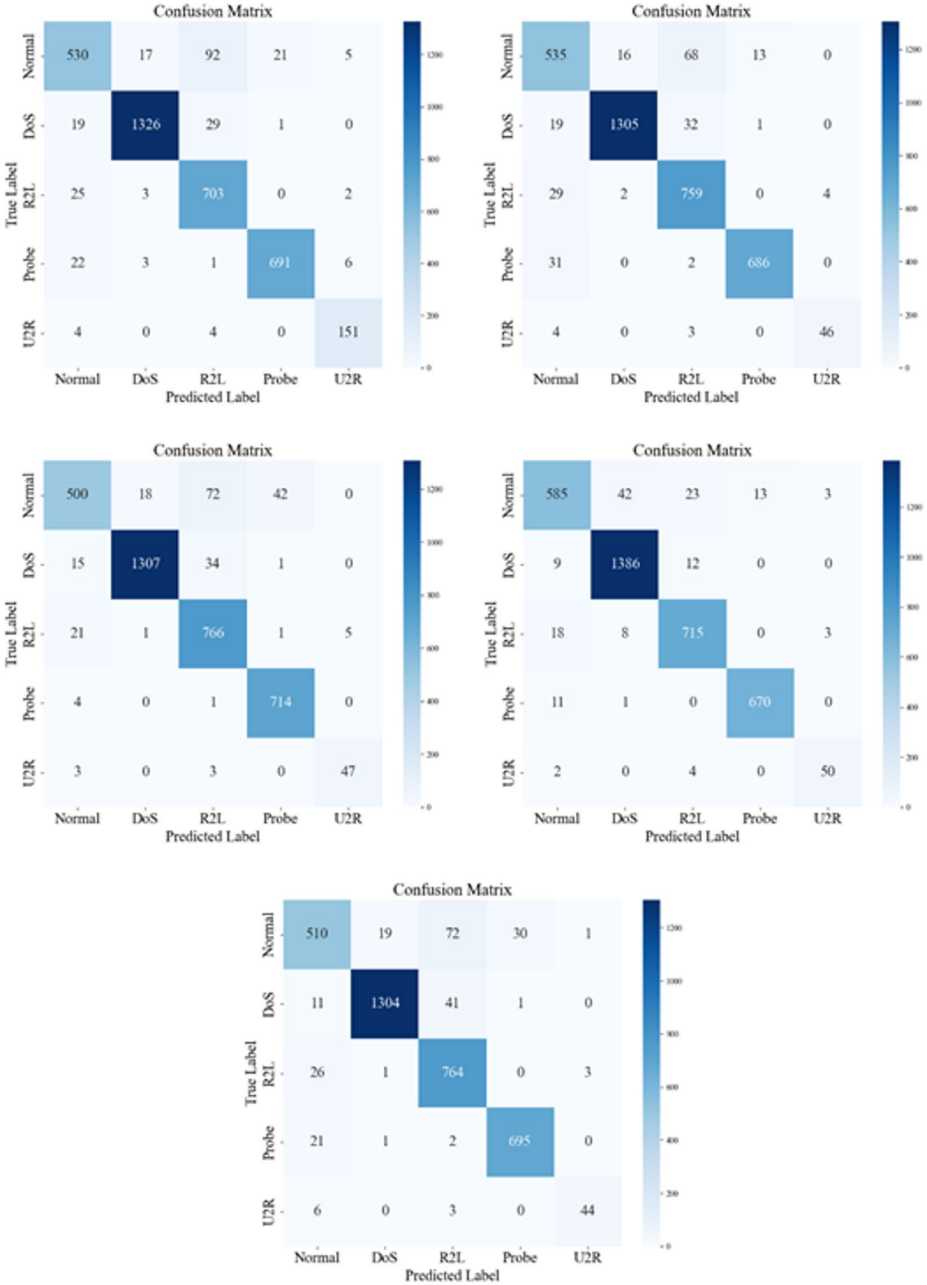
Confusion matrix combined with a heatmap.

To visually present the model performance and data distribution differences, the results are represented using a confusion matrix combined with a heatmap. In this visualization, darker regions indicate higher consistency between predicted and true values, while lighter regions indicate lower consistency. As illustrated in Fig. 10, the darker regions in the confusion matrix of the proposed model are mainly concentrated along the main diagonal, clearly indicating a high degree of alignment between predictions and true labels across all categories. This result further confirms that the proposed model achieves excellent performance in multi-class classification tasks, with high classification accuracy consistent with expectations.

### Ablation study on framework components

To quantify the individual contributions of the proposed components and verify that the performance gains stem from their synergistic effect rather than a single module, a comprehensive ablation study was conducted on the NSL-KDD dataset. We evaluated five degraded variants of the proposed framework: (1) w/o Augmentation: Removing the ADASYN-FLS-SMOTEBoost module, using original imbalanced data; (2) w/o Multi-CNN: Concatenating all features into a single 1D vector and feeding them into a single-branch CNN; (3) w/o FLS Fusion: Replacing the FLS-guided dynamic fusion with static average pooling; (4) w/o HLSTM: Replacing the hierarchical HLSTM with a standard single-layer LSTM; (5) w/o IGWO: Using empirical default hyperparameters (e.g., learning rate = 0.001) instead of IGWO optimization.

**Table 8:** Ablation study of individual components on the NSL-KDD (KDDTest+) dataset.

Model	Variant	Accuracy (%)	Precision (%)	Recall (%)	Macro-F1 (%)
w/o	Augmentation	88.15	89.23	75.34	76.42
w/o	Multi-CNN	89.42	90.11	85.67	86.84
w/o	FLS Fusion	90.18	91.04	86.25	87.51
w/o	HLSTM	89.65	90.56	84.19	85.33
w/o	IGWO	90.72	91.55	87.30	88.02
Proposed (Full Framework)		92.41	93.16	90.98	92.69

As presented in Table 8, removing any core component leads to a distinct performance degradation. The most severe drop in Recall and Macro-F1 occurs in the w/o Augmentation variant, confirming that ADASYN-FLS-SMOTEBoost is crucial for handling class imbalance. Furthermore, the absence of HLSTM significantly impairs the model’s ability to capture long-term temporal attack chains, while the exclusion of FLS Fusion causes feature interference. The full framework achieves the highest performance across all metrics, validating the necessity and strong synergy of the integrated modules.

### One-class detection comparison

Since anomaly detection is an unsupervised learning problem, model evaluation presents certain challenges. To effectively measure and compare the performance of different models, this paper adopts the Area Under the Curve (AUC) as the primary evaluation metric, which is the most commonly used metric for one-class problems^57^. AUC is defined as the area under the Receiver Operating Characteristic (ROC) curve^58^, independent of threshold selection, and can be used to assess the overall performance of a classifier. However, when the sample ratio changes, AUC exhibits low sensitivity to model performance. In highly imbalanced scenarios, the Precision–Recall Curve (PRC) serves as an important metric for evaluating predictive effectiveness. In practical applications, data are often severely imbalanced; PRC helps more intuitively reflect the actual performance of classifiers and guides model improvement and optimization. Therefore, the ROC curve is used to evaluate the overall performance of classifiers in the experiments, while the PRC curve is employed to analyze the classification capability of models on imbalanced data.

**Fig. 11:**
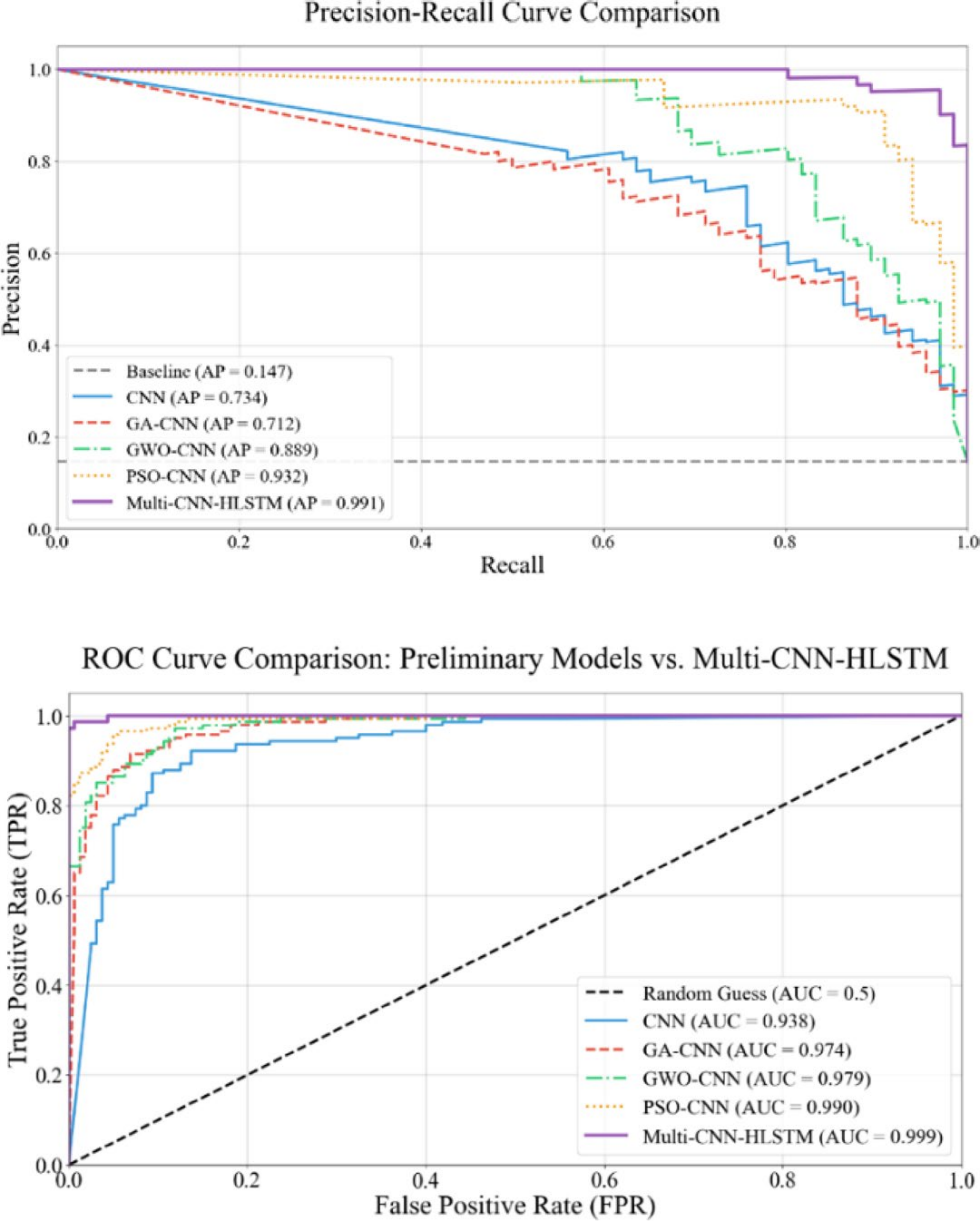
PRC and ROC experience.

**Fig. 12:**
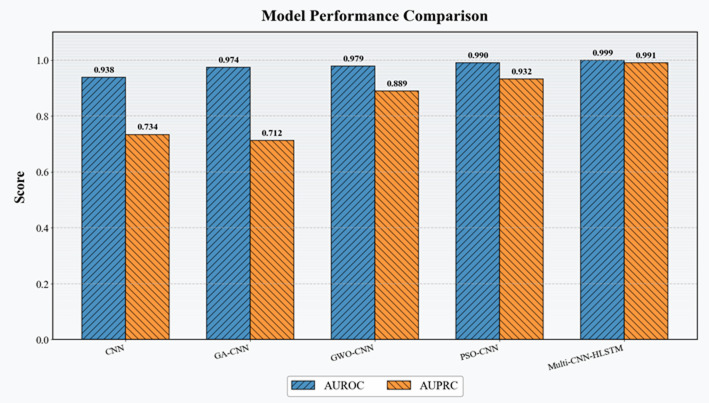
Model performance comparison.

The NSL-KDD dataset contains four types of anomalies, and a one-class dataset can be constructed for each type. In the experiment, any one class is designated as normal samples, while the remaining classes are treated as anomalous samples, thereby generating multiple sets of experimental data. This experiment follows the original train/test split, using only the training set corresponding to the designated normal class for model training. All records are preprocessed: categorical features are first encoded via one-hot encoding, then all feature values are normalized to the [0,1] range using min-max normalization^59^, resulting in the final experimental dataset. Figures 11 and 12 present the experimental results, where Fig. 11 visually demonstrate the overall anomaly detection performance of CNN, GA-CNN, GWO-CNN, PSO-CNN, and the proposed Multi-CNN-HLSTM model through PRC and ROC curves, as well as comparative bar charts of AUPRC and AUROC. As clearly shown in the figures, Multi-CNN-HLSTM achieves the best performance across core evaluation metrics: AUROC reaches 0.999 and AUPRC reaches 0.991, significantly outperforming other comparison models. PSO-CNN and GWO-CNN follow closely; although GA-CNN achieves an AUROC comparable to GWO-CNN, its AUPRC lags noticeably behind. The traditional CNN model performs relatively weaker, with AUROC at 0.938 and AUPRC at only 0.734.

**Table 9:** Detection performance by attack type on the NSL-KDD dataset.

Type	DoS (AUROC/AUPRC)	Probe (AUROC/AUPRC)	R2L (AUROC/AUPRC)	U2R (AUROC/AUPRC)
CNN	0.995/0.991	0.984/0.977	0.938/0.812	0.947/0.825
GA-CNN	0.997/0.994	0.986/0.980	0.974/0.712	0.955/0.841
GWO-CNN	0.998/0.995	0.988/0.982	0.981/0.865	0.959/0.856
PSO-CNN	0.998/0.996	0.987/0.981	0.976/0.932	0.961/0.869
Multi-CNN-HLSTM	0.999/0.998	0.991/0.987	0.999/0.991	0.998/0.990

In the overall detection performance comparison (see Fig. 12), the Multi-CNN-HLSTM framework demonstrates near-saturation advantages in both AUROC and AUPRC metrics, significantly outperforming shallow baseline methods and other optimized deep models. This fully indicates that the proposed method possesses stable and powerful discriminative capability when facing complex traffic scenarios. However, overall performance alone cannot fully reveal the model’s specific behavior across different attack categories. To address this, this paper further decomposes the detection results by attack type (DoS, Probe, R2L, U2R) and presents detailed comparisons in Table 9.

Among the four attack types, R2L anomalies remain the most challenging category. These attacks are often concealed within normal traffic, exhibiting subtle and sparse features, which makes their detection significantly more difficult than for high-frequency, prominent anomalies such as DoS and Probe. In the experimental results, it is clearly observable that all models exhibit relatively low AUROC performance on this category. For example, the traditional CNN achieves only 0.938 AUROC under R2L scenarios, while GA-CNN, despite genetic algorithm optimization, reaches merely 0.974 AUROC. More critically, due to the extreme class imbalance in the test set, the AUPRC metric is further substantially affected. Even PSO-CNN, which performs relatively well, attains only 0.932 AUPRC under R2L and U2R scenarios, while GA-CNN’s AUPRC drops as low as 0.712. This reflects that traditional shallow models and deep models relying solely on evolutionary optimization still perform inadequately when confronting low-frequency, highly stealthy attacks. In contrast, the proposed Multi-CNN-HLSTM framework maintains near-perfect performance even on these high-difficulty anomalies (R2L and U2R), achieving AUROC and AUPRC of 0.999 and 0.991, respectively significantly surpassing shallow baseline models and comprehensively outperforming other deep comparative methods. This result highlights the strong adaptability and robustness of the proposed method in handling highly imbalanced data and complex anomaly types.

Meanwhile, another noteworthy phenomenon was observed in the experiments: in detecting Probe-type attacks, some shallow baseline methods achieved slightly higher AUROC values than certain deep models. For instance, the traditional CNN achieves 0.984 AUROC under Probe scenarios, nearly matching GA-CNN’s 0.986, and even slightly exceeds some deep models in certain statistical learning methods. This suggests that in scenarios such as Probe, where anomaly patterns are clear, features are relatively concentrated, and sample sizes are relatively abundant, shallow models still retain certain advantages. However, from a comprehensive performance perspective, deep models still perform better. Taking AUPRC as an example, CNN achieves only 0.977 under Probe scenarios, whereas GWO-CNN, PSO-CNN, and Multi-CNN-HLSTM reach 0.982, 0.981, and 0.987, respectively demonstrating the advantage of deep architectures in capturing complex dependencies and maintaining stability.

### Forensic analysis of the U2R detection bottleneck

The detection of User-to-Root attacks constitutes a persistent challenge in intelligent intrusion detection, as reflected by the F1-score of approximately 17.87%. A multi-level forensic analysis provides a comprehensive understanding of the factors influencing this performance. Data in Table 10 reveals that U2R encapsulates a variety of stealthy exploits with differing detection complexities. The model demonstrates superior capability in identifying buffer overflow attempts, where temporal signatures in flow duration provide distinguishable patterns. Conversely, detection rates for rootkit and loadmodule exploits remain low because these activities utilize standard system calls. This overlap makes such attacks statistically indistinguishable from authorized administrative sessions at the network level.

**Table 10:** Detailed breakdown of U2R sub-type detection on KDDTest-21.

U2R Attack Category	Total Samples	Correctly Detected	Misclassified as Normal	Individual Recall
buffer overflow	20	12	8	0.600
loadmodule	9	2	7	0.222
rootkit	13	1	12	0.077
perl and others	158	32	126	0.203
Weighted Average	200	47	153	0.235

Class imbalance is managed through the application of cost-sensitive weights within the Focal Loss function. The objective function is formulated as follows:

53 $$\:\begin{array}{c}L=-\sum\:_{i=1}^{N}{\omega\:}_{{y}_{i}}\alpha\:{\left(1-{p}_{i,{y}_{i}}\right)}^{\gamma\:}log\left({p}_{i,{y}_{i}}\right)\end{array}$$

In this expression, the penalty weight $\:\omega\:$ acts as a gradient modulator during backpropagation. By assigning a significantly higher weight to the U2R category ($\:{\omega\:}_{\mathrm{U}2\mathrm{R}}=10\cdot\:{\omega\:}_{\mathrm{N}\mathrm{o}\mathrm{r}\mathrm{m}\mathrm{a}\mathrm{l}}$), the loss contribution of a single missed attack is magnified tenfold compared to a misclassified normal record. This forcing function compels the HLSTM optimizer to prioritize the minimization of U2R empirical risk, effectively shifting the decision boundary deeper into the feature space of the majority class to capture stealthy attack vectors.

While this configuration successfully elevates the recall rate, it introduces an inevitable collateral effect on precision. Because U2R exploits frequently utilize legitimate system calls, their feature representations are often subsumed within the high-density clusters of normal traffic. The heightened sensitivity induced by the penalty weight $\:\omega\:$ causes the model to flag legitimate but infrequent administrative behaviors as potential intrusions. Consequently, the precision for U2R collapses due to the sheer volume of normal traffic outliers that are caught in the widened detection net. This boundary drift constitutes a deliberate architectural choice, ensuring that high-damage, concealed threats are detected even if it necessitates a higher rate of manual audit for false alarms.

**Fig. 13:**
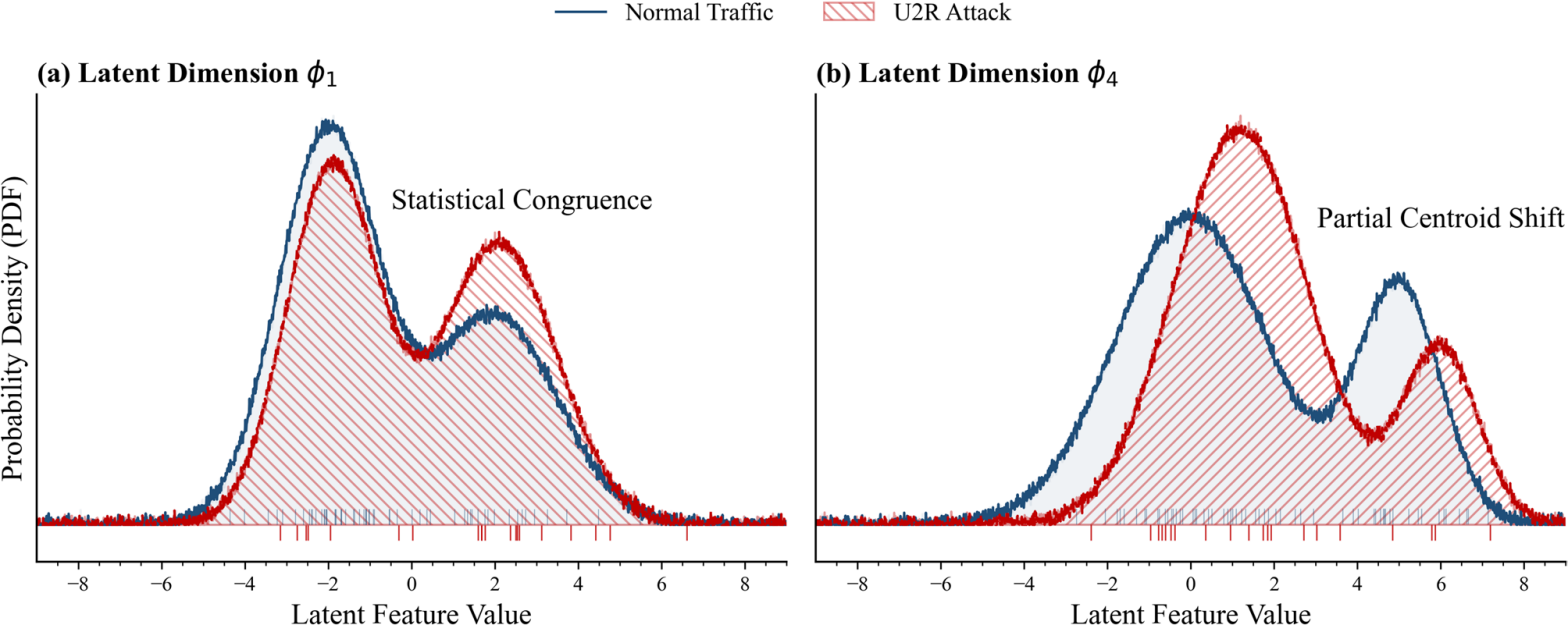
Probability density profiles of latent feature representations for Normal traffic and U2R attacks.

The performance metrics for User-to-Root (U2R) attacks indicate an inherent challenge in flow-based threat modeling, as evidenced by the F1-score of approximately 17.87%. To investigate the statistical basis of this limitation, a comparative probability density function (PDF) analysis was performed across multiple latent dimensions extracted by the HLSTM layer. As illustrated in Fig. 13(a), in certain primary latent projections ($\:{\phi\:}_{1}$), the Normal traffic and U2R attacks exhibit near-complete statistical congruence. While the hierarchical architecture of the HLSTM attempts to isolate adversarial features in other dimensions, such as the marginal centroid shift observed in Fig. 13(b) ($\:{\phi\:}_{4}$), the distribution tails remain largely subsumed within the high-density regions of legitimate sessions.

This pervasive feature overlap confirms the absence of a distinct separation boundary. By assigning a high penalty weight ($\:{\omega\:}_{\mathrm{U}2\mathrm{R}}=10\cdot\:{\omega\:}_{\mathrm{N}\mathrm{o}\mathrm{r}\mathrm{m}\mathrm{a}\mathrm{l}}$) within the cost-sensitive Focal Loss, the model is compelled to sensitize its decision boundary. However, the resulting boundary drift inevitably captures outliers from the massive Normal class, thereby suppressing precision while achieving an actionable recall of 23.5%. This provides a crucial defensive layer against concealed threats previously invisible to statistical learners.

### Evaluation of generalization efficacy on modern threat landscapes

To validate the robustness of the Multi-CNN-HLSTM framework against contemporary network threats, the experimental scope is extended to the UNSW-NB15 dataset. Unlike legacy datasets, UNSW-NB15 encompasses nine modern attack families, including Fuzzers, Backdoors, and Worms, which exhibit higher entropy and more complex statistical distributions. Furthermore, the proposed model is benchmarked against recent state-of-the-art (SOTA) architectures, including TimesNet (2023), a multi-periodicity 2D-variation model, and Swin-Transformer, adapted for one-dimensional sequence modeling.

**Table 11:** Comparative performance on the UNSW-NB15 dataset against SOTA architectures.

Model	Architecture Category	Accuracy (%)	Precision (%)	Recall (%)	Macro-F1 (%)
Swin-Transformer	Self-Attention	84.12	83.30	80.15	81.69
TimesNet (2023)	Multi-periodicity CNN	86.45	87.12	83.40	85.22
Contrastive-NIDS	Self-supervised Learning	84.70	85.90	80.88	83.31
Multi-CNN-HLSTM	Proposed (Hybrid)	88.76	89.11	86.42	87.74

The results in Table 11 indicate that the proposed framework maintains a performance margin of approximately 2.52% in Macro-F1 over the strongest baseline, TimesNet. While Transformer-based models excel at capturing long-range global dependencies through self-attention mechanisms, they frequently encounter semantic dilution when processing heterogeneous network features. Standard attention layers treat disparate dimensions, such as protocol flags and session duration as a uniform sequence, potentially obscuring local correlations within specific feature groups.

The superiority of the Multi-CNN-HLSTM stems from its domain-aware architectural prior. By employing FLS-guided multi-branch convolutional paths, the semantic integrity of basic connection, content, and host-based features is preserved before temporal modeling. This divide-and-conquer strategy proves more effective for modern attacks like Fuzzers, where localized statistical anomalies are more indicative of intrusion than global periodicity. Consequently, the performance lead on UNSW-NB15 confirms that the integration of heterogeneous feature grouping and hierarchical temporal extraction provides a more resilient defense against evolving, high-complexity attack vectors than generic sequence-to-sequence models.

### Computational cost and practical deployability

Beyond classification accuracy, computational efficiency is a critical factor for real-world NIDS deployment. We evaluated the parameter size, training time, and inference speed of the proposed framework and baseline models under identical hardware conditions. Table 12 summarizes the computational cost metrics. The proposed Multi-CNN-HLSTM exhibits a larger parameter footprint (3.82 M) and requires higher training time per epoch (215 s) due to the hierarchical temporal modeling and the IGWO hyperparameter search process. However, the offline training overhead does not impede online operational efficiency. During the deployment phase, the average inference time per sample is approximately 0.15 milliseconds. This inference speed translates to a processing throughput exceeding 6600 network flows per second, which strictly satisfies the low-latency requirements of modern real-time intrusion detection systems. The framework effectively shifts the heavy computational burden to the offline training stage while preserving high-speed deterministic inference.

**Table 12:** Computational cost and efficiency comparison.

Model	Parameters (M)	Training Time (s/epoch)	Inference Time (ms/sample)
CNN	1.25	45	0.08
GA-CNN	1.25	132	0.08
PSO-CNN	1.25	118	0.08
Multi-CNN-HLSTM	3.82	215	0.15

### Validation on contemporary threat scenarios (UNSW-NB15)

To further substantiate the generalization capability and practical relevance of the proposed framework against modern cyber threats such as APTs, Fuzzers, and IoT-based attacks, we extended our evaluation to the contemporary UNSW-NB15 dataset. Unlike NSL-KDD, UNSW-NB15 encompasses 9 modern attack families and reflects complex, contemporary network traffic topologies. We maintained the exact same architecture and compared it against the baseline deep learning models used in previous sections.

**Table 13:** Overall multi-class detection performance on the contemporary UNSW-NB15 dataset.

Model	Accuracy (%)	Precision (%)	Recall (%)	F1-Score (%)
CNN	82.34	83.15	80.05	81.12
GA-CNN	84.51	85.62	81.44	83.25
PSO-CNN	85.12	86.09	82.51	84.03
Multi-CNN-HLSTM	88.76	89.11	86.42	87.94

As shown in Table 13, the proposed framework consistently outperforms existing optimized deep learning baselines on the UNSW-NB15 dataset. The results explicitly demonstrate that the FLS-guided multi-branch feature extraction and hierarchical temporal modeling are not overfitted to legacy data distributions. Instead, the framework maintains robust feature discriminability and high detection efficacy when confronted with modern, high-complexity attack vectors.

## Conclusion and future prospects

This paper addresses the challenges of class imbalance, feature heterogeneity, temporal dependency complexity, and hyperparameter sensitivity in network intrusion detection by proposing an integrated framework that incorporates multiple mechanisms. At the data level, an adaptive oversampling strategy combining ADASYN and Focal Loss–Softmax is employed to strengthen boundary and hard-to-classify samples. At the feature level, a multi-branch CNN is designed, and an FLS-based fusion mechanism is introduced to achieve sample-level dynamic weighting. For temporal modeling, a hierarchical HLSTM is utilized to capture both short-term bursty patterns and long-term latent dependencies. At the optimization level, an improved Grey Wolf Optimizer is introduced to enable efficient hyperparameter search and tuning.

Experimental results demonstrate that the proposed method outperforms mainstream baselines on datasets such as NSL-KDD, particularly in terms of detection rates for rare attack categories and macro-averaged metrics including F1, AUC, and AUPRC. These findings validate the effectiveness and robustness of the framework in complex and imbalanced scenarios. Moreover, the combination of multi-branch CNN–FLS fusion and HLSTM-based multi-scale modeling enhances both interpretability and generalization.

Future research may further integrate self-supervised and contrastive learning to improve cross-domain and zero-day attack detection, as well as incorporate graph neural networks to model associations between traffic and hosts, thereby enabling more precise characterization of complex attack chains. Overall, the proposed approach demonstrates strong potential in both theory and practice, offering a novel perspective for intelligent intrusion detection.

## Data Availability

The datasets used and/or analyzed during the current study are not publicly available, but are available from the corresponding author upon reasonable request.
